# Catching up with drought: law and policy responses in the Netherlands

**DOI:** 10.1007/s10113-025-02449-y

**Published:** 2025-08-20

**Authors:** Max Frederik Wicher Augustijn, Edwin Alblas, Andries Richter

**Affiliations:** 1 Law Group, Wageningen University & Research, Hollandseweg 1, 6706 KN Wageningen, The Netherlands; 2 Environmental Economics and Natural Resources Group, Wageningen University & Research, Hollandseweg 1, 6706 KN Wageningen, The Netherlands

**Keywords:** Drought, Legal framework, Policy mix, Multi-level implementation, Climate adaptation

## Abstract

In recent years, it has become evident that regions with historically wetter climates, such as the Netherlands, are rapidly becoming more prone to drought due to climate change. Given that these regions tend to be accustomed to having sufficient, or even surplus, water, these new climate realities confront policy makers with the need to identify governance approaches to adapt water and land use systems to prevent significant damages to agriculture, water-dependent nature, and drinking water reserves. This research details the complex, evolving landscape of the policies, laws, and instruments in a specific region with a historically wetter climate, namely the Twente region in the Netherlands. Rapid Policy Network Mapping is employed to map the intricate multi-level implementation setting currently in place in the region. Results show that the emphasis in drought governance across European Union and national and regional governance layers is currently still on formulating objectives and developing strategies to adapt to drought. Moreover, the instruments that are currently in place are largely voluntary in nature, which raises concerns about the degree to which the current instrument mix can steer drought adaptation if their uptake is low. More worryingly, this study also identifies several policies, laws, and instruments that could hinder the implementation of drought adaptation measures, due to exemptions in legislation, conflicting policy approaches across governance layers, and concerns over the (behavioral) effects of certain instruments.

**Supplementary Information:**

The online version contains supplementary material available at 10.1007/s10113-025-02449-y.

## Introduction

Droughts have globally become more frequent and hazardous in the past decades due to climate change (Ahmad & Kam 2024; Berbel & Esteban 2019; Jiang & Zhou 2023; Spinoni et al. 2014). Even historically wet regions are increasingly facing droughts, as the consecutive dry years of 2018, 2019, and 2020 in the Netherlands, among others, have recently demonstrated (Brockhoff et al. 2022; Huang & Hartemink 2020; Sweet et al. 2017). Given that these wetter regions are historically speaking accustomed to having sufficient water, or even a water surplus, policy makers in these countries are increasingly confronted with the need to adapt water and land use systems to new climate realities to prevent significant damages to agriculture, water-dependent nature, and drinking water reserves (Brockhoff et al. 2022). As is the case with sustainability transitions in general, such a “drought transition” is multifaceted and requires the adaptation of physical water and land use systems, as well as the adoption of new policies and legislation that can coordinate and enable this systemic change and a wider change in societal attitudes (Barbanente & Grassini 2022; Pahl-Wostl et al. 2010; Turnheim et al. 2015). It is in this context that this research centers on studying drought governance, which is understood as the interaction between public, private, and societal actors in search of means to effectively cope with the current and future occurrence of drought (Bressers et al. 2016b; Brockhoff et al. 2022). Drought itself is conceptualized here as a temporary water shortage condition compared to an average situation, herein referring to a local negative balance between water supply (i.e., from precipitation, water retention, and lateral inflows) and the demand of water-requiring activities (Bressers et al. 2016b; Lloyd-Hughes 2014). Given the diverse sources of water supply and broad range of activities requiring water resources, drought governance entails managing the reciprocal effects of drought and activities related to drinking water, agriculture and horticulture, industrial activity, infrastructure, navigation, and environmental protection (Browne et al. 2016).

Although drought governance is not a novel object of study in historically arid regions, it is still understudied in the context of historically wet countries and regions, especially in comparison to flood protection (Brockhoff et al. 2022). Nevertheless, recent research has provided valuable insights into drought governance in historically wet countries, focusing on transition mechanisms and processes (Brockhoff et al. 2022), assessments of local governance structures (Bressers, Bressers, & Larrue 2016), the fundamental debate regarding water as commodity or common good (Bakker 2014), European Union legislation and Member State interpretation (Keessen & Van Rijswick 2012; Starke & Van Rijswick 2021; Wuijts et al. 2023), and the use of economic instruments (Rey et al. 2019), among others. Although these studies generally touch upon policies, laws, and instruments that are employed by governments, a structured overview of the mix of policies, laws, and instruments in place across governance layers—from supranational to regional layers—, focusing on the *substance*, is missing in the current literature. This is problematic, because climate adaptation governance is often characterized by decentralization, where competences are shared between multiple governance layers (Alblas & van Zeben 2023; Bannink & Ossewaarde 2012; Fermeglia 2023; Hölscher et al. 2019). Generally speaking, the rationale behind adopting such multi-level implementation settings is that they can facilitate policy implementation, provided that policies, laws, and instruments align across governance layers and local conditions are more accurately addressed by regional layers. However, multi-level implementation settings can also hinder policy implementation, when instruments are conflicting across layers, or when steering capacity gaps exist because resource and substantial capacities are not adequately transferred to regional layers, for example (Echeverri et al. 2023; Rogge & Reichardt 2016; Van Der Jagt et al. 2023). In other words, a gap in literature currently exists with regard to complementarities, conflicts, and steering capacity gaps across governance layers for drought adaptation.

This research seeks to fill this gap by providing the first in-depth overview of drought policies, laws, and instruments across governance layers in a historically wet region. To ensure sufficient focus and depth, especially with regard to the implementation of law and policy on regional levels, this research zooms in on the Twente region in the Netherlands, which has proven to be particularly vulnerable to drought in recent years. This vulnerability is due to an interplay of factors that can also be seen in other parts of the world, including changes in precipitation patterns due to climate change (i.e., more and longer periods of abnormally dry weather), the porosity of its sandy soils, and the water system, which has been heavily modified to enhance its drainage capacity to facilitate intensive agriculture (Brockhoff et al. 2022; Huang & Hartemink 2020; *Nationaal Deltaprogramma 2024*, 2023; *The Netherlands National Strategic Plan CAP 2023–2027*, 2023; van Hussen et al. 2019). As such, this research aims to answer the following question: “which mix of policies, laws and instruments is currently in place across governance layers that address drought challenges in the Netherlands, focusing on the Twente region?” As an additional exploratory objective, this study furthermore aims to gain insights into the spatial specificity of policies, laws, and instruments, and their alignment with geospatial conditions. This objective is motivated by the aforementioned premise that decentralized governance enhances policy implementation when local circumstances are effectively addressed at regional levels (Alblas & van Zeben 2023; Ohlhorst 2015; van der Horst 2006). By mapping local conditions alongside the spatial targets of relevant policies, laws, and instruments, and identifying both strengths and limitations, this study aims to highlight opportunities to strengthen policy implementation.

## Theoretical and analytical framework

This overview study is grounded in theoretical conceptions stemming from two strands of literature: multi-level implementation studies and policy mix studies.

First, drawing from multi-level implementation studies, this research is interested in “the process of interpretation of superordinate law by actors who are embedded within distinct and multiple contexts” (Thomann & Sager 2017). In many countries, implementation is characterized by decentralized governance processes, where regional governments, which have shared competence in spatial planning and issues concerning the environment, are regulatees that translate domestic law into regional regulations and policy (Fermeglia 2023). As mentioned in the introduction, policy implementation can be facilitated by multi-level implementation settings if policies, laws, and instruments align across governance layers and local conditions are more accurately addressed by regional layers, or hindered if instruments are conflicting across layers or when steering capacity gaps exists because resource and substantial capacities are not adequately transferred to regional layers (Alblas & van Zeben 2023; Bannink & Ossewaarde 2012; Fermeglia 2023; Hölscher et al. 2019). It is in this context that Gollata and Newig (2017) advocate the closer study of the (degree of) cooperation and coordination occurring between different layers of governance, from supranational (i.e., transcending national governments) down to regional layers (i.e., provinces), manifested in the forms of both vertical and horizontal governance relationships. Thus, following literature from multi-level implementation studies, this research aims to study the extent to which the Twente region has caught up with increasing drought risks by identifying which drought legislation, policies, and instruments are currently in place across multi-level governance layers.

Second, building on policy mix studies, this study is particularly interested in the coordination between law, policies, and instruments to evaluate how these may operate in complementary or synergistic ways, or how they may undermine one another by serving conflicting interests (Echeverri et al. 2023; Rogge & Reichardt 2016; Van Der Jagt et al. 2023). This study understands law, which can refer to an act, regulation, or directive, as a set of legally binding rules that are formulated and enforceable by government actors, that seek to convey the rights and obligations that are being created (Bodansky 2016; Heyvaert 2018). If adopted with regulatory intent, legislation can steer a target group’s behavior to serve a goal which is deemed in the public interest. Policies, in contrast, are understood as non-binding guidelines that are formulated, in the first place by government actors, to steer decision-making and outline actions to solve specific problems affecting, directly or indirectly, societies across different periods of times and geographical spaces (Ruiz Estrada 2011). Instruments then, are understood as the means by which the rules and guidelines of law and policy are implemented, herein referring, among others, to economic instruments, such as subsidies, and command-and-control instruments, such as water extraction regulations (Leshinsky & Legacy 2016; Taylor et al. 2012). It is in the context of these definitions that legislation and policy can both set goals and provide instruments to achieve goals. While recognizing that drought governance is a broad concept, the main focus of this study is thus on studying the “formal” rules in place and their application (i.e., implementation) in the form of subordinate legislation, policy guidelines, and policy instruments. In adopting this framing, informal aspects of governance, such as social norms and (private) governance by companies and other civil actors, are considered to be outside of the scope of this study (Böcher 2012).

This study largely adopts a “traditional” typology to study the policy mix, by categorizing identified policy instruments as command-and-control, economic, informational, and cooperative instruments (Böcher 2012; Gunningham & Sinclair 1999; Taylor et al. 2012). In this study, command-and-control instruments refer to instruments that “impose legally binding obligations or restrictions on the behavior of firms or individuals” and, given their legally binding nature, are found in legislation (Gunningham & Sinclair 1999; Taylor et al. 2012). Economic instruments, on the other hand, are understood as voluntary instruments that provide specific pecuniary incentives (or disincentives) such as taxes, charges, and subsidies (Borrás & Edquist 2013; Gunningham & Sinclair 1999). Informational instruments are also considered voluntary instruments and encompass instruments that aim to reduce knowledge asymmetries between actors, such as regulator and regulatees, small and big firms, society, and business or buyers and suppliers, by providing information in the form of education, knowledge sharing, monitoring, and disclosure (Gunningham & Sinclair 1999). Finally, cooperative instruments rely on coordination mechanisms between actors to establish voluntary measures (Böcher 2012). While this typology may insinuate that instrument types are mutually exclusive, this study subscribes to the notion that certain instruments can be associated with multiple types (Rogge & Reichardt 2016). For example, subsidies that are conditional on meeting legally binding requirements can be considered economic instruments with command-and-control elements.

This typology of instruments is primarily adopted because past research has provided valuable insights on the strengths and weaknesses in policy mixes by employing this typology, which can be used to interpret observations. For example, command-and-control instruments are often deemed to act in complementary fashion with economic or cooperative instruments to stimulate performance “beyond compliance” (Gunningham & Sinclair 1999; Taylor et al. 2012). Similarly, economic instruments can also facilitate compliance to command-and-control instruments when, for example, the costs of adopting more sustainable practices are relatively high compared to maintaining existing practices (Borrás & Edquist 2013; Gunningham & Sinclair 1999). Informational instruments, on the other hand, are deemed to synergize with all types of instruments, given their aim to reduce knowledge asymmetries (Gunningham & Sinclair 1999). Authors furthermore argue that instrument types vary in the degree to which they can reliably steer actors to behave in a certain way (Böcher 2012; Gunningham & Sinclair 1999; Taylor et al. 2012). For example, voluntary instruments, such as economic and informational instruments, can have limited effects on the behavior of actors if their uptake is low (Böcher 2012).

Adding onto the existing policy mix literature, this study includes two instrument types in the typology stemming from planning literature, namely project-based instruments and vision statements, to obtain a more complete view on the governance of the physical and infrastructural dimensions of sustainability transitions (Hurlimann & March 2012; Leshinsky & Legacy 2016; Stead 2021; van Dijk 2005). Project-based instruments are defined as specific activities and projects to be undertaken directly by governments that concern the adaptation of physical space and infrastructure, such as replacing waterworks, vegetation in natural areas, or reprofiling water trajectories (Hurlimann & March 2012; Leshinsky & Legacy 2016; Stead 2021; van Dijk 2005). Although often overlooked in policy mix literature, the adaptation of public infrastructure is often a key part of environmental problem-solving approaches by governments (Hurlimann & March 2012). By means of illustration, in the case of flood management, an overview of governance responses would remain incomplete if project-based instruments such as the construction of dykes, dams, and flood barriers were not included. Vision statements, on the other hand, are understood as general statements of desired future outcomes (Hurlimann & March 2012; Stead 2021). Vision statements are often central to non-binding policy and provide broad overall directions and motivations to public and private actors, without necessarily being tied to specific actions. Vision statements were included in this study to capture the non-binding guidelines that steer policy implementation considering the uncertainties that exist (i.e., technological developments, the severity of climate change impacts) and to identify alignments and incongruencies between vision statements across layers and the instruments to implement them. The inclusion of vision statements can furthermore shed light on the degree to which policy is developed for a particular issue, as their formulation is often part of the process of agenda-setting, which commonly occurs in the early stages of policy cycles (Albert et al. 2021; Hurlimann & March 2012; Pahl-Wostl et al. 2010).

## Material and methods

### Study area: the Twente region in the Netherlands

**Fig. 1:**
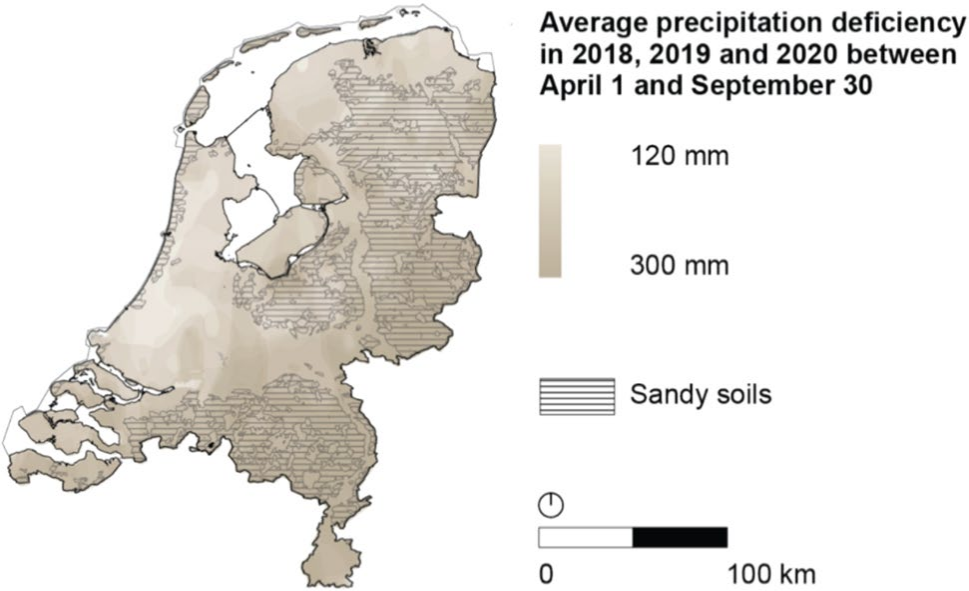
Precipitation deficiencies in the Netherlands during the consecutive dry years of 2018–2020 were highest in the Eastern and Southern sandy soil regions

The Netherlands is a historically wet country grappling with the need to adapt to drought. During the droughts of 2018, 2019, and 2020, drought measures were mostly reactive and temporary in nature (Projectteam Droogte Zandgronden Nederland 2021). Depending on the region, measures included temporary increases of upstream water levels, irrigation bans, and increasing non-local water intake (i.e., supplying water systems with water from outside the region, for example, from the Rivers Rhine and Meuse). Post hoc evaluations suggest that these reactive measures ultimately showed limited and temporary effectiveness and yielded significant costs for farmers and society (Projectteam Droogte Zandgronden Nederland 2021). The impact was especially pronounced in sandy soil regions, which constitute 48% of the country’s soils and are particularly sensitive to droughts due to their porosity and low soil organic matter content, as can be seen in Fig. 1 (Silvis et al. 2016). Various sources claim that 2018 drought damages amounted to €820–€1,400 million to agriculture (i.e., crop damages) and €500 million to nature (Beleidstafel Droogte 2019; *The Netherlands National Strategic Plan CAP 2023–2027*, 2023; van Hussen et al. 2019). Due to concerns about future droughts, governments in the Netherlands have started to look for structural measures that encompass a shift in water management approaches, by moving away from a water system that is modified to enhance water drainage (i.e., to facilitate intensive agriculture and cope with water surplus) to a system that is better capable of retaining water and by reducing water demand across sectors (Bressers et al. 2016a, b; Brockhoff et al. 2022; Projectteam Droogte Zandgronden Nederland 2021; Provincie Overijssel 2022).

While the challenges faced are not unique to the Netherlands, there are certain peculiarities about the Dutch way of governing water. Similar to other European countries, regional governments in the Netherlands have competence in governing territorial issues such as the protection of the environment and spatial planning (Fermeglia 2023). However, the regional governance layer of water authorities (*waterschappen*) is unique to the Netherlands, even when compared to their Belgian and Surinamese namesakes. Water authorities in the Netherlands are democratically elected governments that, under the auspice of the national government, are responsible for managing regional water bodies, dealing with the flow of watercourses, drainage, water collection, flood, erosion prevention, irrigation, phreatic ground water management, and the provision of potable water. Water authorities’ administrative borders tend to follow the geophysical boundaries of one or multiple watersheds and tend to cross provincial and municipal borders (Wuijts et al. 2023). In the end, what truly sets Dutch water authorities apart from other forms of regional water authorities is the unique combination of their wide management responsibilities, high degree of autonomy in implementing climate adaptation policy, their competence to impose taxes, their democratic election, and their historical significance in the political and spatial development of the country (Havekes et al. 2024; Kastelein 2024). As such, studying Dutch water authorities can offer unique insights from the perspective of a multi-implementation setting where local water authorities have a relatively high degree of autonomy (i.e., financial and in terms of climate policy implementation) and experience a relatively high degree of legitimacy in water management in light of their historical importance (Guerrin et al. 2014; Kastelein 2024).

Next to the water authorities layer, three more governance layers are involved in water governance in the Netherlands. The European Union is mainly involved in setting objectives for water bodies and resources, the conservation of (water-dependent) nature and (agricultural) land use in its Member States. The national government then implements these objectives with, among others, national legal, planning, and economic instruments, and is directly involved in the management of the major water bodies in the country, such as the Rhine River and Lake IJssel. Provincial governments are primarily responsible for regional spatial planning, formulating land use and water extraction regulations and for the management of acquifers. The municipal government layer was excluded from the analysis, even though municipalities play important roles in managing sewage systems, enforcement (i.e., of water and land use regulations), and as shareholders in drinking water companies. However, they have limited competences in setting “the rules of the game” in terms of water management and land use (Gemeentewet 2025; i.e., Verordening kwaliteit leefomgeving gemeente Enschede 2023, 2025). In addition, most municipalities are still in the process of formulating their implementing policy and legislation, specifically the Municipal Environment and Planning Plan (i.e., Omgevingsplan gemeente Enschede 2024), because the Dutch Environment and Planning Act (Omgevingswet 2024), which is the law that primarily steers land and water use in the Netherlands, only entered into force in 2024.

**Fig. 2:**
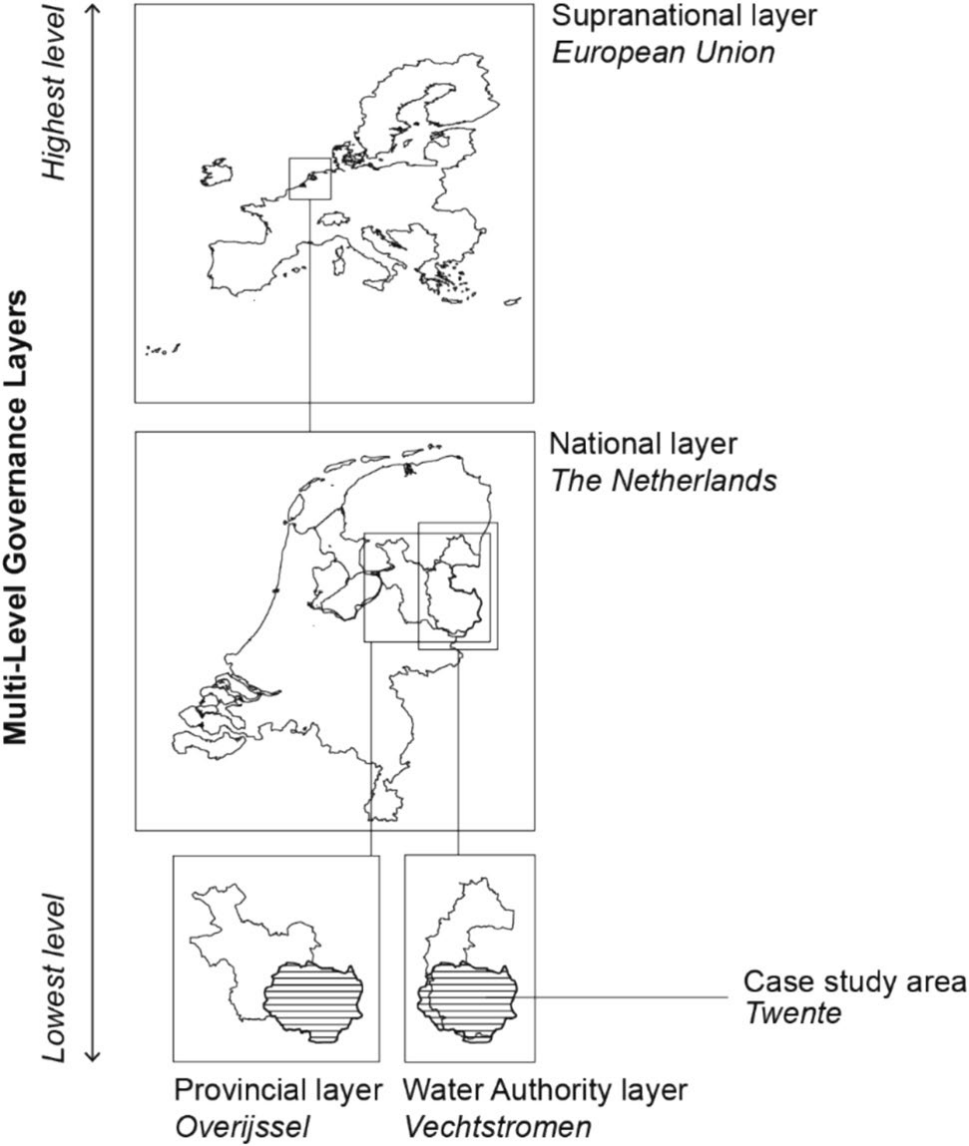
Four governance layers involved in the multi-level implementation setting of the case study area Twente: The European Union, the Dutch national government, the Overijssel Province, and the Vechtstromen Water Authority

The “Twente” region in the East of the Netherlands was selected as the study area, which is situated within the administrative boundaries of the Overijssel Province and the Vechtstromen Water Authority, as can be seen in Fig. 2. This study area was chosen for two reasons. First, the region consists almost entirely of sandy soils that are vulnerable to drought. Second, the region is topographically and geomorphologically diverse, containing both topographically lower, and therefore wetter areas (i.e., stream valleys), and drier areas that are topographically higher (i.e., moraines and sand ridges), as well as areas that cannot be supplied with non-local water intake. This diversity was deemed relevant because drought policies, laws, and instruments are ideally tailored to these conditions to maximize effectiveness (Projectteam Droogte Zandgronden Nederland 2021).

### Data collection method and instrument categorization

A three-stepped Rapid Policy Network Mapping method was used to identify and study relevant policies, laws, and instruments on European Union, national, provincial, and water authority layers (Alexander et al. 2015; Bainbridge et al. 2011). As a first step, a snowballing approach was used in which each policy instrument was used as a referral source to identify related instruments. The Water Framework Directive, Common Agricultural Policy, Climate Law, and Birds and Habitats Directives were selected as starting points for the snowballing process, given that these laws are often regarded to be the most fundamental instruments for water and land use governance in the European Union (Bakker 2014; Bastmeijer et al. 2021; Berbel & Esteban 2019; Bressers et al. 2016b; Rey et al. 2019). Drought instruments presented in the documents were identified by utilizing the following search queries: “drought,” “water scarcity,” “freshwater,” “water availability,” “water quantity,” “climate adaptation,” “water stress” and their equivalents in the Dutch language. In certain cases, search queries merely found results in the explanation of the enactment of the identified document or in the title of a section, while the relationship between the instruments and drought was not mentioned explicitly in the text, as is the case with instruments that address the water retention capacity of soils through the maintenance of soil organic matter, for example. In such cases, academic literature and research referenced in the studied document were consulted to verify whether the instrument addressed drought. Documents were obtained through the following official data portals: (1) EUR-Lex (https://eur-lex.europa.eu) for the European Union layer; (2) Officiële Bekendmakingen and Overheid.nl (https://www.officielebekendmakingen.nl and wetten.overheid.nl) for the national layer; (3) Overijssel.nl (https://www.overijssel.nl) for the provincial layer; and (4) Vechtstromen.nl (https://www.vechtstromen.nl) for the water authorities layer. As a second step, the aforementioned search queries were executed on the data portals to ensure all relevant policies, laws, and instruments were obtained. Then, as the third step, policies, laws, and instruments were mapped to visualize linkages between legal and policy documents across governance layers in the multi-level implementation setting.

To study and map the policy instrument mix, this study categorized instruments as command-and-control, economic, cooperative, informational, project-based instruments and vision statements, in accordance with the definitions outlined in the theoretical and analytical framework section.

### Geospatial data and visualization

To map geospatial conditions, administrative boundaries, and spatial policy targets, geospatial data was collected and processed. All geospatial datasets, predominantly obtained in Geopackage, TIF, GeoJSON, and Shapefile formats, were loaded into the open-source Geographic Information Systems software QGIS for data management, processing, and visualization. Geospatial datasets were obtained from Dutch public data sources. Processing of the datasets was mostly limited to the use of filter functions to highlight features relevant for drought governance. A full overview of the utilized datasets and data processing procedures is provided in Appendix A of the supporting information.

## Results

**Fig. 3:**
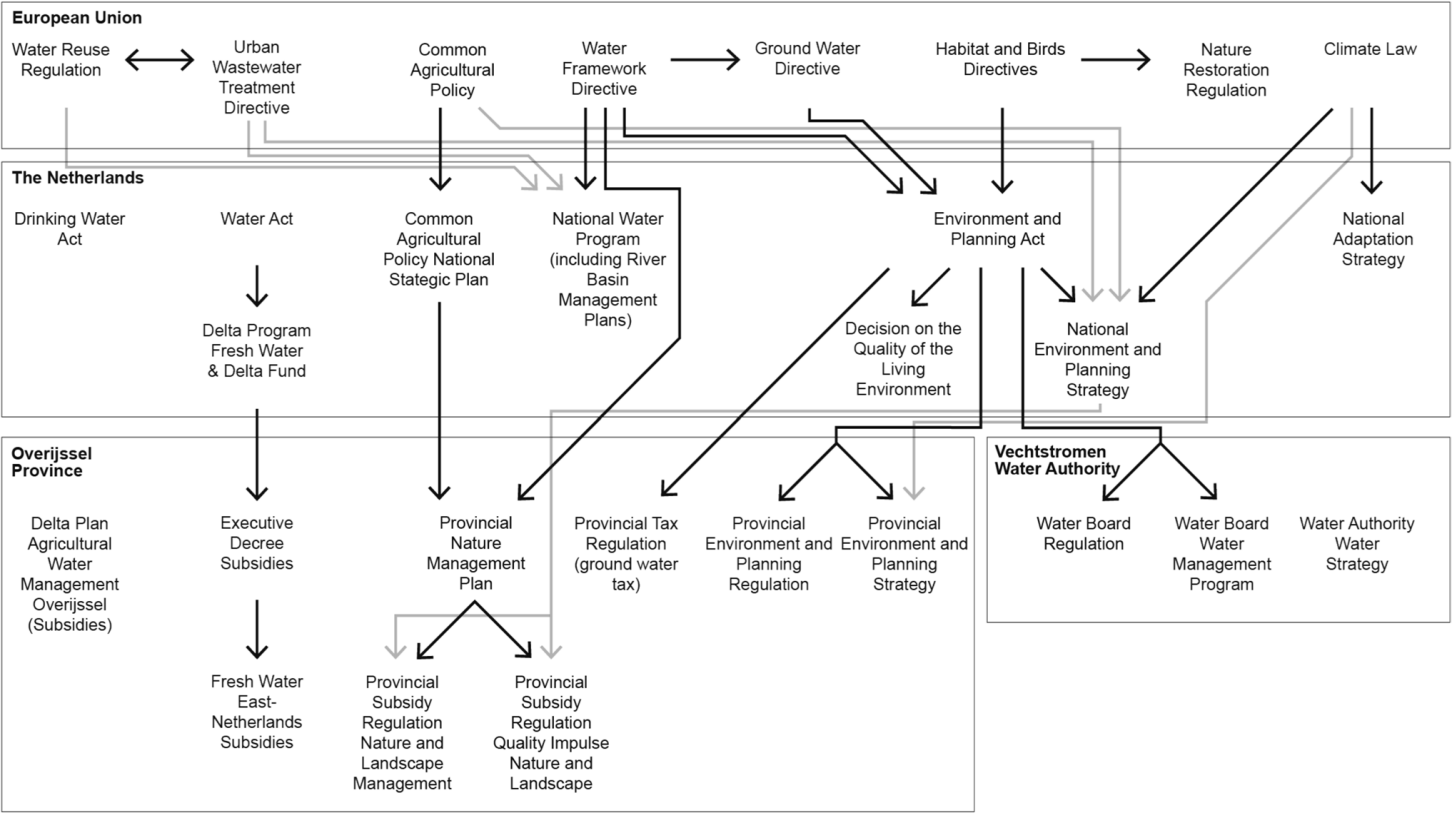
Visualization of drought policies and legislation across governmental levels. The lines in between documents illustrate the referrals between policies and legislation. Some links are gray to enhance legibility when lines cross

This chapter presents an overview of the drought policies, laws, and instruments that were identified. This chapter is structured by moving from the highest governance layer, the European Union level, to the regional layers, being the provincial and water authority layers. Figure 3 visualizes the policies, laws, and instruments that were identified and how they interrelate across layers in the multi-level implementation setting. An overview of the translations and abbreviations of policies, laws, and instruments can be found in Appendix B, and an extensive discussion and analysis of policy, laws, and instruments can be found in Appendix C.

### Supranational layer: European Union

As stated in article 192(2b) of the Treaty on the Functioning of the European Union (2016), the European Parliament, the Economic and Social Committee, and the Committee of the Regions are responsible for adopting measures affecting the quantitative management of water resources. Although there is currently no dedicated “Drought Directive,” or comparable legislation, in place in the European Union, elements of drought governance are embedded in various pieces of legislation on the European Union level (Hervás-Gámez & Delgado-Ramos 2019; Jancewicz 2024).

Aiming to “establish a framework for the protection of inland surface waters, transitional waters, coastal waters, and groundwater,” which includes mitigating the effects of floods and droughts (Bastmeijer et al. 2021; Copetti & Erba 2023; article 1e of Directive 2000/60/EC, 2000), command-and-control instruments targeting drought challenges are prevalent throughout the Water Framework Directive (Directive 2000/60/EC, 2000). In essence, the Directive requires Member States to include drought-related measures in their River Basin Management Plans, which Member States must establish to outline their strategy for six-year periods to protect and, where necessary, restore water bodies. Drought measures can be found in provisions that concern balancing water extraction and recharge, protecting water-dependent ecosystems, implementing adequate water pricing, and adapting agricultural practices by introducing crops that require less water.

Water pollution provisions, such as those laid down by the WFD and the adjacent Groundwater Directive (Directive 2006/118/EC, 2006), are often said to address drought indirectly. That is, during droughts, pollutant concentrations in water bodies rise due to the reduced water volume available for dilution, and after droughts, pollutants that have accumulated in the soil can run off into water bodies, hereby breaching WFD water quality requirements (Berbel & Esteban 2019; Furusho et al. 2016). However, this view can be somewhat contrasted with article 4(6) of the Water Framework Directive (Directive 2000/60/EC, 2000), which pertains that a temporary deterioration in the status of water bodies is not in breach with water quality requirements if this is a consequence of prolonged droughts, on the conditions that Member States define indicators for such unforeseen circumstances, that all practicable measures are taken with the aim of restoring the water body and that the effects of these circumstances and the measures taken are included in the next version of the River Basin Management Plan (Starke & Van Rijswick 2021). Although such exemptions can make goal achievement more manageable, they have also been criticized in the literature for enabling Member States to lower the ambitions of the Water Framework Directive (Boeuf et al. 2016). An exemption that sparks a similar concern is the exemption in article 11(3e), which allows Member States to exempt from “controls, authorization or registering water extractions or impoundments which have no significant impact on water status.”

The Common Agricultural Policy is historically aimed at ensuring a stable food supply and safeguarding farmer incomes (article 39 of Treaty on the Functioning of the European Union 2016). Recent reforms, however, have integrated climate change mitigation, adaptation, and sustainable resource management objectives (Heyl et al. 2020; i.e., articles 5, 6 and 105 of Regulation (EU) 2021/2115, 2024). The Common Agricultural Policy provides several economic instruments for farmers that concern drought adaptation, such as direct income support that is conditional on, among others, water extraction authorization, crop rotation, and (arguably) soil organic matter preservation measures; voluntary eco-scheme payments aimed at climate adaptation; sector-specific payments for interventions in tangible and intangible assets including insurance against adverse climatic events and advisory services; and Rural Development Fund payments for climate-related management commitments, for farmers in areas with area-specific constraints and for climate adaptation–related investments in irrigation, risk management tools (which includes insurance against adverse climatic events that is not sector-specific), and knowledge exchange.

The Birds and Habitats Directives (Directive 2009/147/EC, 2009); Directive 92/43/EEC, 1992) mandate the protection of species and habitats of communal importance, among others, through the designation of a network of protected sites called “Natura 2000,” by Member States. Although both Directives do not contain any notion of drought or climate change, researchers have argued that, in accordance with Water Framework Directive provisions on the designation of areas of special protection, water-dependent nature (i.e., bogs and wet heath) should receive protection from drought to conserve habitats and species directly dependent on water, hereby promoting drought adaptation in and around protected habitats (Stein et al. 2016).

The Nature Restoration Regulation (Regulation (EU) 2024/1991, 2024) aims to restore ecosystems to ensure the recovery of biodiverse and resilient nature in the European Union and, in doing so, aims to contribute to the European Union’s climate change mitigation and climate change adaptation objectives. The Nature Restoration Regulation touches upon drought adaptation by requiring Member States to enhance the quality, quantity, and connectivity of freshwater, agricultural, and forest ecosystems and to restore the natural connectivity of rivers. More specifically, the restoration of freshwater ecosystems often implies an improvement of hydrological conditions within and around natural areas, as can be further substantiated by the measures listed in the annexes of the Nature Restoration Regulation. Furthermore, restoring the connectivity of freshwater ecosystems and rivers can also contribute to enhancing drought resilience by enhancing the flow of water during droughts. This enhanced water flow, in turn, can be used by natural vegetation and for irrigation, enhances groundwater recharge, and mitigates biodiversity decline by reducing the accumulation of pollutants in water bodies and by allowing aquatic species to migrate to safer areas during drought (Lake 2003; Reich & Lake 2015; Sarremejane et al. 2022; Thieme et al. 2024). However, concerns can be raised over the exemptions listed in article 4(14 and 15), which allow for derogations by Member States in improving habitat quality in natural areas outside of Natura 2000 if the deterioration is the consequence of natural disasters, climate change or the situation of a project of overriding public interest in the area. Member States are mandated to develop National Restoration Plans to outline measures to restore habitats, yet due to the fact that the Regulation only recently entered into force, the Dutch National Restoration Plan is not yet published.

The European Climate Law (Regulation (EU) 2021/1119, 2021) can be considered relevant to drought adaptation given that it aims to enhance the adaptive capacities and resilience of Member States to climate change—alongside aiming to achieve climate neutrality in 2050 within the European Union. Three command-and-control instruments can be identified that address drought: the establishment of the European Scientific Advisory board that raises awareness and stimulates dialogue and cooperation between scientific bodies in the European Union on the topic of climate change; the adoption of a European Union strategy on climate change adaptation by the Commission, and the adoption and implementation of National Adaptation Strategies by Member States, which include measures to adapt to climate change, and; the establishment of multi-level climate dialogues between local authorities, civil society organizations, business communities, investors, stakeholders, and the general public to discuss the achievement of climate policies.

Finally, two relatively new pieces of legislation were identified that address drought with provisions on water reuse. The revised Urban Wastewater Treatment Directive (Directive (EU) 2024/3019,  2024) sets the legal framework for the collection, treatment, and discharge of urban wastewater and the discharge of biodegradable wastewater from certain industrial sectors. Most concretely addressing drought is article 15(1), which states that “Member States shall systematically promote the reuse of treated wastewater from all urban wastewater treatment plants where appropriate, especially in water-stressed areas, and for all appropriate purposes.” Strategies to implement water reuse are featured in Integrated Urban Wastewater Management Plans, which Member States must establish soonest by 2033 for agglomerations of 100,000 population equivalents and above. The Water Reuse Regulation (Regulation (EU) 2020/741, 2020), as the name suggests, is concerned with facilitating the uptake of water reuse by Member States whenever it is appropriate and cost-efficient. The Regulation addresses drought in article 6, which allows for agricultural water reuse on the condition that water quality conditions are met, and article 9, which pertains that water reuse should be a subject of awareness campaigns in Member States.

### National layer: the Netherlands

Legal frameworks that concern the physical environment have been undergoing considerable change in the Netherlands with the Environment and Planning Act (Omgevingswet 2024) entering into force in January 2024. The Act consolidates 26 individual laws concerning, among others, water management, nature conservation, agriculture, and spatial planning into a single act with supplementary laws. A main aspect of the Act is that it requires the national, provincial, water authority, and municipal governments to define their respective strategies (*Omgevingsvisie*) and regulations (*Omgevingsverordening*) on spatial planning and environmental management (articles 2.4–2.6 and 3). At the national level, the government is required to formulate two documents: a National Strategy on Spatial Planning and the Environment (*Nationale Omgevingsvisie*) and a Decision on the Quality of the Living Environment (*Besluit Kwaliteit Leefomgeving*).

The Environment and Planning Act (Omgevingswet 2024) addresses drought in several instances with command-and-control instruments. First, it requires national and regional layers to include a hierarchy in societal and ecological necessities in their regulations that is decisive in the allocation of surface water during scarcity, and that may be applied to ground water as well. Second, the Act contains provisions on water extraction activities, mandating that Provincial Environment and Planning Regulations, Municipal Environmental Plans, and Water Authority Management Plans must include provisions on water extraction activities to prevent water scarcity, which are enforced with Environmental Permits (*Omgevingsvergunning*). Finally, the Environment and Planning Act states that groundwater taxes based on the volume extracted can be established by provinces, which may differ for extraction activities in and outside of groundwater protection areas used for drinking water production. Thus, considering the contents of these provisions, the Environment and Planning Act can be regarded essential for the implementation of Water Framework Directive provisions on water extraction and pricing on the national layer.

**Fig. 4:**
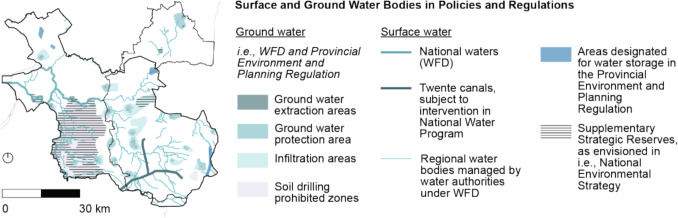
The Water Framework Directive (WFD) mandates Member States to determine protected surface and ground water bodies. The map shows water bodies protected under the WFD, as well as water bodies and areas that are subject to various other national and regional policies and regulations

**Fig. 5:**
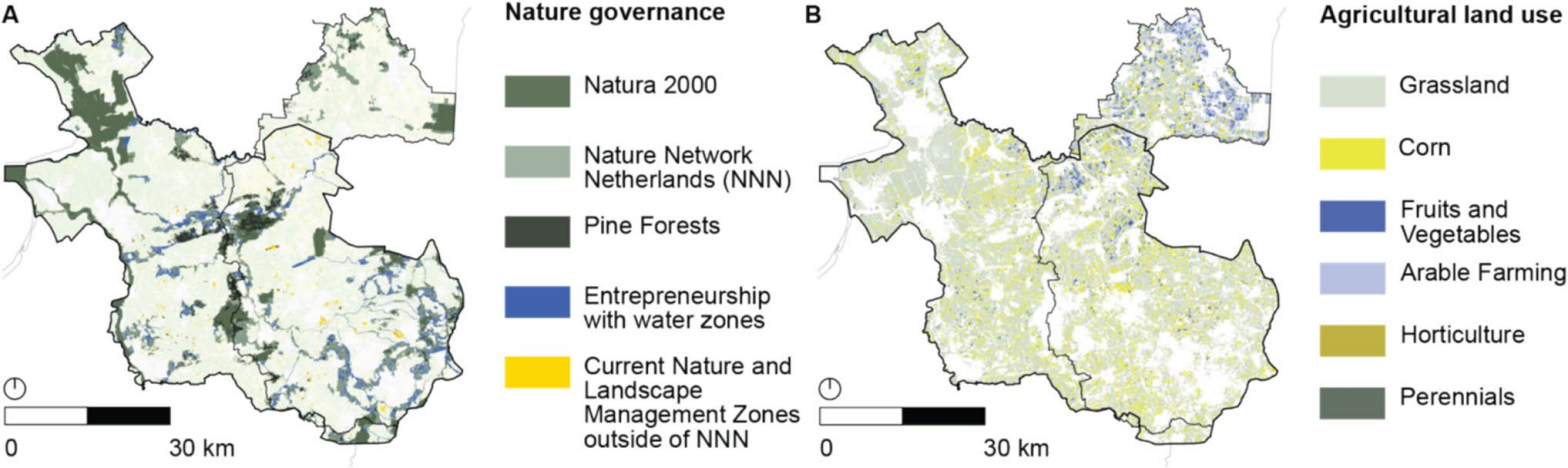
Panel **A** shows nature protected under the Natura 2000 and Nature Network Netherlands (NNN) regimes, and highlights pine forests, which are envisioned to be converted to deciduous forests or heath. Provincial subsidies for “Entrepreneurship with Water” are provided in areas close to NNN areas and in several stream valleys. The provincial Nature and Landscape Management subsidies are currently granted in NNN areas and several smaller areas outside of NNN. Panel **B** shows agricultural land use. Grass and corn are widespread throughout the region, reflecting the presence of livestock farming. Currently, provincial Nature and Landscape Management subsidies are provided to shift from corn to cereals. The fruits and vegetable sectors, which receive sector-specific payments for climate adaptation interventions in the National Strategic Plan, and arable farming are predominantly situated in the North-East of the Vechtstromen region. Horticulture and perennial farming are scarce in the region

The Decision on the Quality of the Living Environment (Besluit Kwaliteit Leefomgeving 2024) provides command-and-control instruments on the national level to safeguard the quality of the living environment, which includes safeguarding reliable drinking water supplies and the protection of natural values. As a first measure relating to drought, and in line with the Environment and Planning Act, it specifies water allocation priorities during scarcity, consisting of four levels: (1) the highest priority is to allocate water to ensure the stability of flood defenses, to prevent land subsidence and permanent damage to nature, followed by; (2) allocating water for drinking water and energy production; (3) allocating water for temporary irrigation of capital-intensive crops and industrial process water, and finally; (4) allocating water to other uses, including inland shipping, fisheries, and less vulnerable agriculture and nature. In addition, the Decision states that rules can be added to the water allocation hierarchy in Provincial Environment and Planning Regulations (*Provinciale Omgevingsverordening*) for regional waters and ground water. Regional governments furthermore have to include provisions in their respective Environment and Planning Regulations to preserve water conditions in Nature Network Netherlands areas, which are nationally protected natural areas that include Natura 2000 areas, and provisions to ensure that Environmental Permits (*Omgevingsvergunning*) are only issued for activities that do not adversely affect Water Framework Directive designated water bodies, and are compatible with water scarcity prevention objectives. Figure 4 shows ground and surface water bodies that are protected under the Water Framework Directive and Fig. 5 shows Natura 2000 and Nature Network Netherlands areas.

Although the Environment and Planning Act consolidates a considerable number of environmental laws, two separate pieces of legislation are still (partially) in place that concern water governance: the Drinking Water Act (Drinkwaterwet 2009) and the Water Act (Waterwet 2024). The Drinking Water Act contains a single provision on drought: “the owner of a drinking water company takes all suitable measures to ensure sufficient drinking water supply to meet future demands in their distribution area.” The Water Act, on the other hand, addresses security of water supplies and water safety by requiring the formulation of the Delta Program (*Deltaprogramma*), which outlines water management measures to ensure water safety and water supply for a period of six years.

Finally, a series of non-binding policies and investment plans were identified on the national layer, which are mandated by the European Union and national legislation discussed previously. In terms of (non-binding) investment plans, the Delta Plan Freshwater (*Deltaplan Zoetwater 2022–2027*, 2021) and the National Strategic Plan of the Netherlands (The Netherlands National Strategic Plan CAP 2023–2027, 2023) present a significant number of investments in drought adaptation measures such as reprofiling water trajectories and adapting drainage and irrigation systems, insurance against adverse climatic events (as part of the Common Agricultural Policy support for sector-specific interventions for fruits and vegetables sectors and risk management tools), land use change (i.e., transforming pine forests, as depicted in Fig. 5, into heath or deciduous forests, subsidies for fiber crops), and knowledge exchange initiatives. Non-legally binding vision statements outlined in the National Water Program (Nationaal Water Programma *2022–2027*, 2022), which includes the River Basin Management Plans, and the National Adaptation Strategy, mandated by the European Union Climate Law, emphasize research and the development of national policies for water use and current and future water availability. In contrast, the National Environment and Planning Strategy (*Nationale Omgevingsvisie (NOVI)*, 2020) presents non-binding vision statements that seem less focused on research and more action-oriented, envisioning the implementation of local drought solutions, aligning land use with water availability (i.e., with European Union Common Agricultural Policy support), creating Supplementary Strategic Supplies (*Aanvullende Strategische Voorraden*) for groundwater and climate buffers around protected nature, and reducing water consumption of civilians and companies. The envisioned Supplementary Strategic Supplies are visualized in Fig. 4, and the climate buffers are envisioned around the protected nature shown in Fig. 5.

### Provincial layer: Overijssel

Since the implementation of the Environment and Planning Act (Omgevingswet 2024), key documents for the regulation of the physical environment on the provincial layer are the Provincial Environment and Planning Strategy (*Provinciale Omgevingsvisie*) and the Provincial Environment and Planning Regulation (*Provinciale Omgevingsverordening*). Zooming into the study area, the Environment and Planning Strategy and Regulation of the Overijssel Province were studied.

The Provincial Environment and Planning Regulation (*Omgevingsverordening Overijssel* 2024f) addresses drought with several command-and-control instruments related to spatial planning and water management. In terms of spatial planning, the Provincial Environment and Planning Regulation requires municipalities to include provisions on the requirements for spatial developments in their Environmental Plans (*Omgevingsplan*), which are enforced with Environmental Permits (*Omgevingsvergunning*). Relating to drought, Municipal Environmental Plans should only permit developments that contribute to a climate-robust water system or the maintenance and enhancement of ecological values in particular areas, namely water storage zones, “areas with specific requirements” (i.e., stream valleys), “entrepreneurship with nature and water” zones (which are situated around natural areas), and protected and unprotected natural areas, as visualized in Figs. 4 and 5. For all spatial developments in general, the Regulation furthermore contains a provision that requires developments to align with the Provincial Environment and Planning Strategy. Nevertheless, the actual requirements for spatial developments remain unspecific in the Regulation, as quantitative target values or permitted land use are not included.

**Fig. 6:**
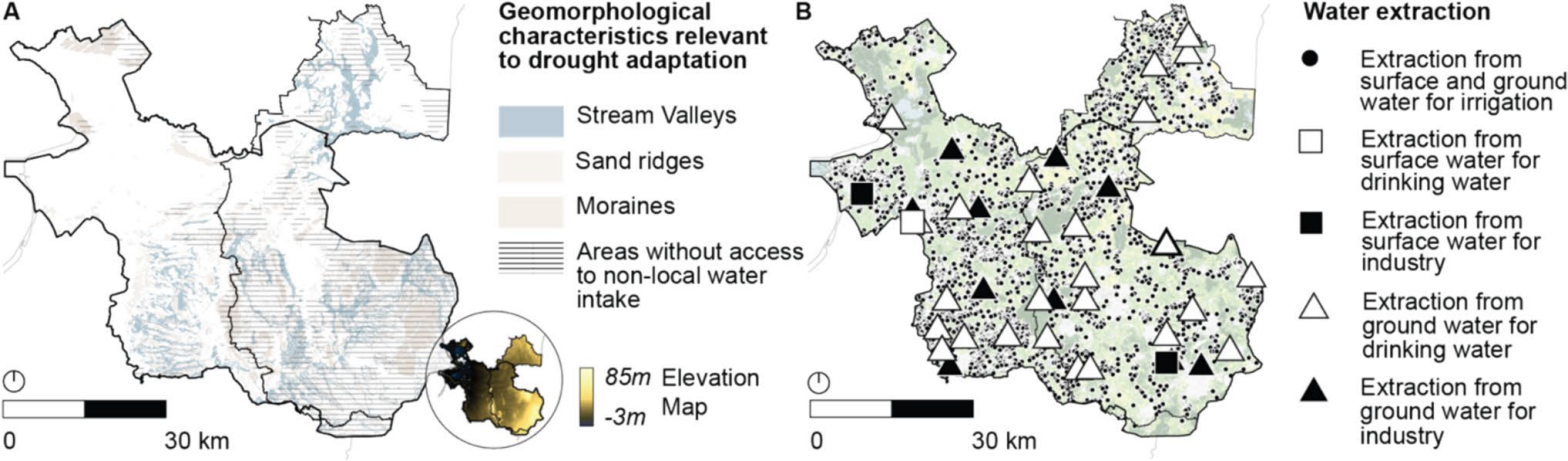
Panel **A** shows geomorphological characteristics that are targeted by drought policies and legislation. Panel **B** shows water extraction activities for irrigation, industry and drinking water production. As can be seen, water extraction activities for industry and drinking water are often located close to urban areas (light gray), and, perhaps more worrying, often in close proximity to nature. Irrigation systems furthermore seem less prevalent on the higher and more drought-prone South-Eastern part of the study area

In terms of water management, the Provincial Environment and Planning Regulation (*Omgevingsverordening Overijssel* 2024f) includes provisions on water extractions. More specifically, it requires that Water Authority Regulations (*Waterschapsverordening*) to only allow water extraction and infiltration volumes between 50,000 and 150,000 cubic meters per year if a permit has been issued or if the extraction activity is reported in advance. Furthermore, permits for extraction and infiltration volumes exceeding 150,000 cubic meters per year for industry and drinking water production are issued by the province, and extractions below 50,000 cubic meters are currently unregulated—which is permitted under the Water Framework Directive’s exemption on authorizing water extractions “which have no significant impact on water status.” Current water extraction activities are shown in Fig. 6. As can be seen, water extraction activities for irrigation are less prevalent in the (drier) East of the study area and more worrisome—yet understandable given the relatively high availability of water at these locations—water extraction activities for industry and drinking water production are often located in close proximity to natural areas. Lastly, the Regulation introduces deviations from the national water allocation priorities, mainly in the fourth and fifth levels priority. Among others, priorities such as flushing pollutants and salinization are added to the hierarchy, and priorities such as fisheries and water recreation are omitted.

The Provincial Environment and Planning Strategy (*Fundament voor de Nieuwe Omgevingsvisie *2022; *Concept Nieuwe Omgevingsvisie *2024g) contains spatially targeted, yet unspecific and non-binding vision statements, such as reducing evapotranspiration (which may imply land use change) and delaying run-off on topographically higher moraines and capturing peak rainfall in topographically lower areas that cannot be supplied with non-local water intake. As can be seen in Fig. 6, Twente consists mostly of topographically higher soils that cannot be supplied by non-local water intake.

Finally, a significant number of voluntary economic instruments are provided in the Freshwater Eastern Netherlands (Uitvoeringsbesluit subsidies Overijssel 2022, 2024a), Delta Plan Agricultural Water Management (*Deltaplan Agrarisch Waterbeheer Overijsselse Maatregelen* 2023), and Nature Management Plan subsidy schemes (*Natuurbeheerplan 2025 Provincie Overijssel* 2024b; *Subsidieregeling kwaliteitsimpuls natuur en landschap Overijssel 2024* 2024c, *Subsidieregeling natuur- en landschapsbeheer Overijssel 2024* 2024d) to incentivize, among others, the adoption of water retention measures, more efficient irrigation systems, and land use change. These economic instruments could be regarded complementary to the command-and-control instruments of the Provincial Environment and Planning Regulation as they may financially enable the envisioned drought adaptation measures orincentivize drought adaptation measures “beyond compliance” (Gunningham & Sinclair 1999; Taylor et al. 2012). In terms of land use change, subsidies are, among others, offered for land use change in stream valleys and of cornfields, which are widespread throughout the region, as can be seen in Figs. 5 and 6. This implies that a large area is eligible for these subsidies, which in turn raises concerns about the potential subsidy burden if (hypothetically speaking) all land users were to be supported. Besides subsidies, the Provincial Tax Regulation (*Belastingverordening *Overijssel 2024e) contains provisions on ground water extraction charges that apply to actors extracting more than 100,000 cubic meters per year.

### Water authority layer: Vechtstromen

Since the introduction of the Environment and Planning Act (Omgevingswet 2024), water authorities are required to formulate two documents: Water Management Plan (*Waterbeheerprogramma*) and Water Authority Regulation (*Waterschapsverordening*). The Water Management Plan describes the Water Authority’s (non-binding) strategy for a time horizon of five years and the Water Authority Regulation contains legally binding provisions, among others, on water extraction activities. Additionally, some water authorities have formulated a Water Strategy (*Watervisie*) to outline the long-term strategy of the water authority for the next thirty years. However, this document is not legally mandated by any law or regulation.

It was found that the Vechtstromen Water Authority Regulation (Waterschapsverordening Waterschap Vechtstromen 2024) and Water Vision (*Watervisie 2050 Waterschap Vechtstromen*, 2020) align closely with command-and-control instruments and vision statements of other governance layers. Most concretely, the provisions on water extraction in the Water Authority Regulation correspond with provisions in the Provincial Environment and Planning Regulation (*Omgevingsverordening Overijssel* 2024f). In addition, the Regulation also shows alignment with other governance layers with provisions on irrigation bans during periods of drought and in areas within 200 m of water-dependent nature, and prioritizing wastewater reuse before discharge. Similarly, the long-term vision statements in the Water Vision, such as increasing the water retention capacity of water and land use systems, largely reflect the vision statements of the National and Provincial Environment and Planning Strategies (*Nationale Omgevingsvisie (NOVI)*, 2020; Provincie Overijssel 2022, 2024).

A deviation and several complementarities with respect to the other governance layers were observed in the Water Management Plan (*Waterbeheerprogramma 2022–2027 Waterschap Vechtstromen*, 2021). The deviation lies in the water authority’s intention to maintain an approach for the next five years where water levels are set to accommodate the desired land use in flat and sloped areas, seemingly regardless of whether this increases drought vulnerability. This approach seems opposite of the envisioned approach on national and provincial layers, which aim to align land use with the local water availability. In terms of complementarities, on the other hand, it was observed that the Water Authority is more inclined to use instruments that are collaborative, informational, and project-based in nature than other governance layers. More specifically, the collaboration track contains informational instruments (i.e., awareness campaigns) aimed at stimulating the uptake of subsidies, efficient water use and land use change, and cooperative instruments aimed at seeking alignment with provinces, municipalities, and drinking water companies on topics such as securing a climate-robust drinking water supply and joint implementation agendas. Although these measures are already established through participation processes, and the Water Authority aims to continue implementing them, these measures are non-binding and lack detailed budgets and timelines. As such, in addition to featuring informational and cooperative elements, these measures should be considered vision statements.

## Conclusions and discussion

**Fig. 7:**
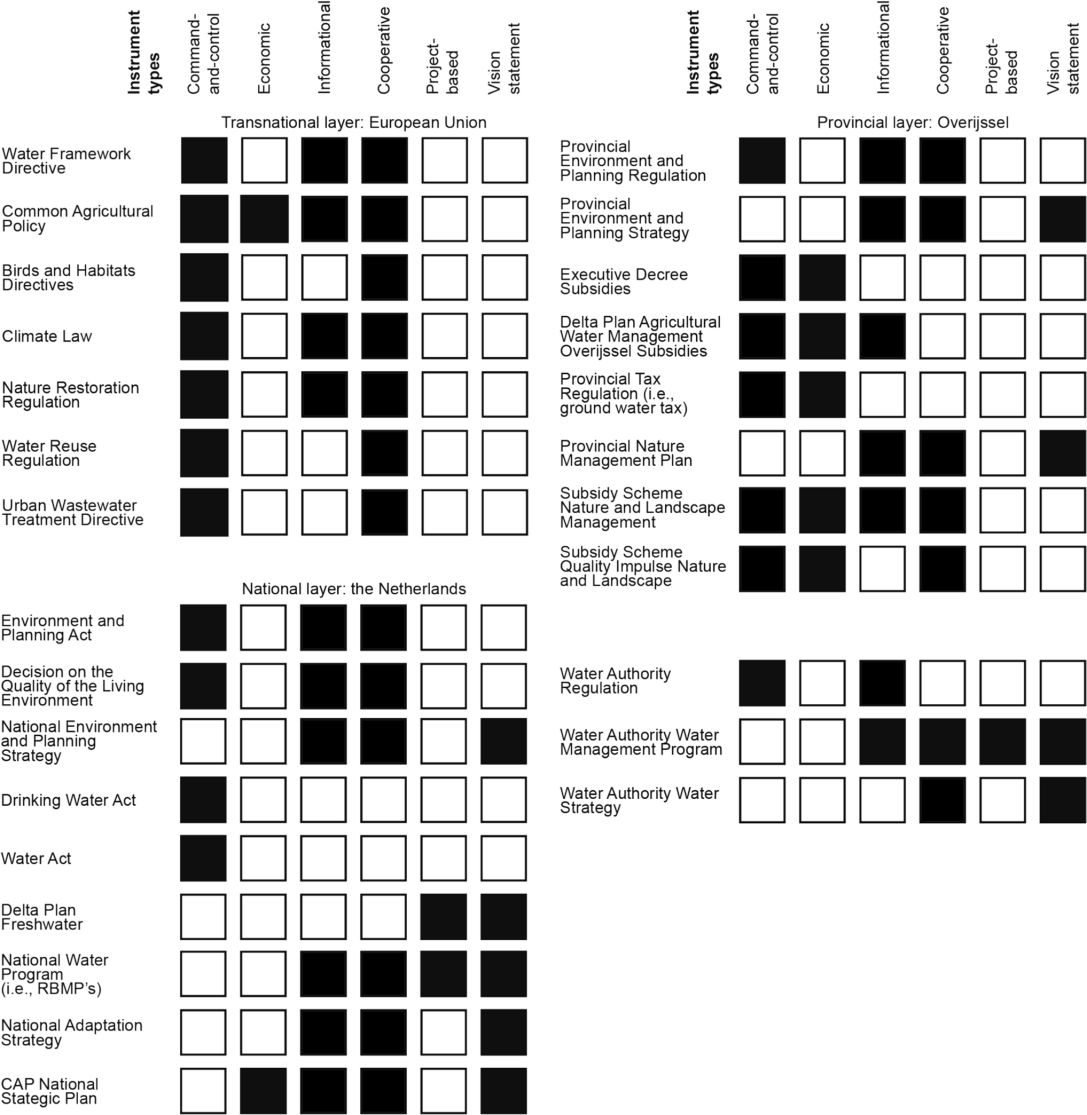
Overview of the policy instrument mix in the multi-level implementation setting. A black fill indicates that elements of an instrument type are present in a policy or law. Elements of informational and cooperative instruments are often found in policy and legislation in the form of stimulating information gathering (i.e., monitoring) or dissemination, and cooperation between government authorities and/or actors in society, respectively. Similarly, economic instruments tend to incorporate command-and-control elements by being conditional on meeting specific thresholds or fulfilling obligations

This research aimed to investigate to what extent the Netherlands has “caught up” with drought by providing an overview of the policies, laws, and instruments that are currently in place to address drought in the multi-level implementation setting of the drought-prone region of Twente. Based on the results outlined in the results chapter, five overall conclusions are formulated, as presented in the next paragraphs. These conclusions are followed by a discussion of the limitations of this study and suggestions for future research. A graphic overview of the identified instruments is provided in Fig. 7.

First, in light of the objective to catch up with the current status of drought governance in a historically wet country like the Netherlands, it can be concluded that, although drought governance is starting to receive more attention in recent years across governance layers, this is still in an early stage. More specifically, it was found that older policies and legislation, from before the drought of 2018, rarely address drought directly (i.e., by explicitly mentioning any of the employed search criteria such as “drought” as an objective in the description of the instrument), whereas more recent policies and legislation tend to address it more often directly. For example, on the national level in the Netherlands, policies and legislation have emerged since 2018 that address drought directly, such as Delta Plan Freshwater from 2022 (*Deltaplan Zoetwater 2022–2027*, 2021), which provides a significant number of investments in physical water system assets and land use change. In contrast, policies and legislation published before 2018, such as the National Adaptation Strategy from 2016 (*Nationale Adaptatie Strategie*, 2016) and the Birds and Habitats Directives (Directive 2009/147/EC, 2009); Directive 92/43/EEC, 1992), often do not mention drought at all. Furthermore, in line with existing research, it can be argued that drought policy making is still at an early stage, as governments seem to focus on formulating vision statements and mostly adopt “softer” instruments that are voluntary and informational in nature (Albert et al. 2021; Hurlimann & March 2012; Pahl-Wostl et al. 2010).

Second, throughout governance layers, concerns are raised about the extent to which certain policy instruments contribute to, or even impede, drought adaptation, such as the financial support for insurance against climatic events, the permittance of temporary breaches in water quality due to drought, and the lack of instruments for water extractions below 50,000 cubic meters per year. Zooming into these concerns, existing literature is for example divided about the effects of insurance against climatic events on farmers’ investments in climate adaptation interventions (Kraehnert et al. 2021). On the one hand, such insurance schemes have been noted to mitigate the economic effects of adverse climatic events, hereby increasing the availability of funds that can be invested in climate adaptation interventions. On the other hand, is has also been suggested that insurance against adverse climatic events can reduce the degree to which actors are willing to adapt to drought, as it may be easier to accept drought-induced damages than to adapt to drought. With regard to the absence of monitoring, authorization, and taxation of water extractions below 50,000 cubic meters per year, a key issue that can be identified is that small water extractions, such as those by citizens for garden sprinklers, remain unregulated. Several water authorities in the Southern provinces of Brabant and Limburg (i.e., outside the study area) have recognized this issue and have implemented reporting obligations to cover these smaller water extractions in recent years (van Houtert 2024). Although this seems a favorable development in terms of ensuring sustainable water use, concerns can still be raised. First, concerns can be raised over the effect that a reporting obligation will have on reducing water extractions, since the reporting obligation is not accompanied by any form of disincentive or quantitative restriction (Provincie Brabant 2024; Verordening grondwaterheffing Noord-Brabant 2023, 2024). Second, provisions for water extractions below 50,000 cubic meters per year are still absent in other water authorities outside of Brabant, which may point to the need for similar regulatory changes on the higher governance layers—i.e., the national layer and possibly the European Union layer, considering notable exemptions on water extraction controls in the Water Framework Directive—to harmonize water extraction legislation. Third, surface water extractions are not covered by this reporting obligation. Reflecting the effects of the now-abolished Dutch groundwater tax, this might ensue an increase of surface water extractions, hence raising doubts with regard to the effect that such a reporting obligation may have on the overall reduction of water extractions (Schuerhoff et al. 2013). Finally, concerns can be raised over the feasibility of monitoring and enforcing provisions on small water extractions. Although there is currently no view on the total number of small water pumps in use, media estimate that the number is in the thousands, considering that 15,000 citizens reported their ground water pumps in less than a year since the registry opened (van Houtert 2024).

Third, the implementation of drought adaptation policies, in particular those concerning land use change, currently relies significantly on voluntary instruments, such as subsidies. Command-and-control instruments exist, but mostly concern ground water protection areas, nature conservation, and the authorization of water extraction. Voluntary instruments that provide strong incentives (e.g., taxes) are often deemed politically unattractive, hindering the implementation of drought adaptation policies (Ejelöv & Nilsson 2020; Sari et al. 2023; Taylor et al. 2012). This raises the question how to increase acceptability and support for more stringent, and potentially more effective instruments, such as crop restrictions in key water retention areas, higher water extraction taxes or taxes for water extractions below 50,000 cubic meters per year.

Fourth, although the majority of instruments and objectives (i.e., groundwater protection areas, water pricing, land use subsidies, climate buffers) tends be integrated in a consistent manner across governance layers, a notable deviation between governance layers was observed: opposite to national and provincial layers, the Vechtstromen Water Authority layer envisions to maintain a management approach in flat and sloped areas where the water system is modified to accommodate the desired land use. Cases of policy incoherence have been found to pose several risks to policy implementation, including causing uncertainty and conflicts over goal achievement among actors (Bressers et al. 2016a). Researchers furthermore attribute policy incoherence to various worrisome reasons, such as insufficient interaction between government layers, knowledge asymmetries, different perceptions of the problem, and conflicting interests (Bressers et al. 2016a; Echeverri et al. 2023; Rogge & Reichardt 2016). Contrasting with these views however, the literature also suggests that policy incoherence can be deliberate and beneficial in multi-level implementation settings. More specifically, cases of policy incoherence should not be viewed in isolation, but rather in a dynamic perspective, by taking into account that subordinate governance layers can deliberately deviate from policy of higher governance layers according to the opportunities of the local context in the current situation (Bressers et al. 2016a).

Fifth, the governance layers involved in drought adaptation tend to have their own roles and scopes in drought adaptation and therefore utilize different types of instruments and address different aspects of drought adaptation. Most notably, it was observed that the provincial layer is the only governance layer that formulates planning regulations for spatial developments, whereas water authorities, who are more directly involved with local water users and physical water infrastructure, employ more informational, cooperative, and project-based instruments. The implications of this observation are twofold. First, and perhaps unsurprisingly, governance layers provide complementary instruments, reflecting their delegated competences and responsibilities, which is generally perceived as beneficial to governance systems (Echeverri et al. 2023; Gunningham & Sinclair 1999). Second, and more worryingly, water authorities might lack command-and-control instruments to steer spatial developments, especially in comparison to provincial governments, which is concerning considering the duty of care of water authorities over phreatic groundwater and regional surface water resources.

Four limitations can be identified with regard to this study. The first limitation regards the three-stepped Rapid Policy Network Mapping method that was employed to identify policies, laws, and instruments relevant to drought. This approach was chosen to highlight the horizontal and vertical linkages between policies, laws, and instruments, which aligns with the focal point of multi-level implementation studies. However, due to the complex and dynamic nature of the multi-level implementation setting, there is a chance that potentially relevant instruments were overlooked, as this method is arguably better equipped to map vertical linkages rather than horizontal ones. Especially policy or legislation that has entered into force after the adoption of primary legislation such as the European Water Framework Directive (Directive 2000/60/EC, 2000) or the Dutch Environment and Planning Act (Omgevingswet 2024) can be easily overlooked as they are often not cited. The third step of the snowballing approach, where search queries were entered on governmental search portals to identify policies, laws, and instruments, was adopted as part of the methodology to address this issue. Although it is still impossible to ensure that the employed search terms were exhaustive, this approach seemed the most appropriate to identify policies, laws, and instruments. A second limitation of this research was that it was limited to studying a single region in the Dutch context. While this allowed for a thorough analysis, a comparative study could have yielded valuable insights into the differences in governance approaches that exist as a result of the discretion that lower governance layers have in integrating legislation. Third, as mentioned in the methodology chapter, the municipal governance layer was excluded from analysis due to its limited role in rule setting for water and land use management in the Netherlands—as compared to provinces and water authorities—and the fact that the key instruments on the municipal governance layer (i.e., *Omgevingsplan*) are still under development. As such, knowledge gaps remain with regard to the instruments that municipalities employ to address drought within multi-level implementation settings. Fourth, the focus of this research has been specifically on how drought management is governed in existing legislation across governance layers. As a result, the legal configuration of Intergovernmental Oversight (*Interbestuurlijk Toezicht*) was not subject to analysis. More specifically, the Netherlands has a system of multi-level Intergovernmental Oversight, which means, in short, that higher governmental levels can exercise oversight over lower levels. The national government oversee tasks of the provinces (article 253 of the Provinciewet 2025; article 121c of the *Wet Revitalisering Generiek Toezicht*, 2012). In turn, national and provincial governments can supervise the performance of tasks by municipalities (article 124e of the Gemeentewet 2025) and water authorities (article 2.18 of the Omgevingswet 2024; article 158 of the Provinciewet 2025). Of particular relevance to drought governance is the fact that provinces hold significant responsibility in the intergovernmental supervision of water infrastructure that is not managed by the national government.

Following from the conclusions and limitations, seven suggestions for future research are formulated. First, stemming from the observation that drought governance in the study area relies significantly on voluntary instruments, future research could study the acceptability, uptake, and application of voluntary instruments to gain a better understanding of the current effect of these voluntary instruments and how to potentially improve current drought governance approaches. Second, following the observed policy incoherence on the water authority layer, and given that policy incoherence can have a multitude of causes and consequences, future research could study the causes and consequences of policy incoherence of this particular case. Such research might contribute valuable insights to the body of literature associated with policy coherence and shed light on the manner by which multi-level implementation structures can be potential sources of improvement or obstacles for policy implementation (Alblas & van Zeben 2023). Third, future research on the Dutch context could study the competences of water authorities in relation to their duty of care, given that the results suggest that water authorities might experience steering capacity gaps in terms of spatial planning. Fourth, given the limitations of the three-stepped Rapid Policy Network Mapping method employed in this study, future research could complement this approach with a validation exercise involving policy makers or practitioners. This would help assess both the completeness of the mapped governance framework and the extent to which the identified policies, laws, and instruments reflect realities in the field. Fifth, stemming from the limitation that this research studied a single region, a suggestion for future research is to conduct comparative studies on the policy mixes in place for drought in multi-level implementation settings. Such studies could focus on comparisons between European Union Member States or Dutch regions, or with multi-level implementation settings outside of the European Union, such as in Eastern Canada and Northeastern United States, which are historically wet regions that have experienced an increased prevalence and severity of droughts in recent years (Jain et al. 2024; Sweet et al. 2017). Sixth, given that the municipal governance layer was excluded from the analysis of this study, a recommendation for future research is to investigate drought governance on the municipal layer in multi-level implementation settings. Finally, considering that this study did not examine the role of Intergovernmental Oversight (*Interbestuurlijk Toezicht*) in drought governance, a future qualitative study could assess how and whether Intergovernmental Oversight is used in practice in the implementation of drought policy and legislation.

## Supplementary Information

Below is the link to the electronic supplementary material.

Supplementary file1 (DOCX 65 KB)

## Data Availability

No new data were created or analyzed during this study. All sources of the data supporting the findings are cited within the article and its supplementary materials. For further information or data-related inquiries, please contact the corresponding author, Max F.W. Augustijn.

## References

[CR1] Ahmad DM, Kam J (2024) Disparity between global drought hazard and awareness. NPJ Clean Water 7(1):75. 10.1038/s41545-024-00373-y

[CR2] Albert C, Brillinger M, Guerrero P, Gottwald S, Henze J, et al. (2021) Planning nature-based solutions: principles, steps, and insights. Ambio 50(8):1446–1461. 10.1007/s13280-020-01365-1 33058009 10.1007/s13280-020-01365-1 PMC8249551

[CR3] Alblas E, van Zeben J (2023) Collaborative agri-environmental governance in the Netherlands: a novel institutional arrangement to bridge social-ecological dynamics. Ecol Soc 28(1):art28. 10.5751/ES-13648-280128

[CR4] Alexander KA, Potts TP, Freeman S, Israel D, Johansen J, et al. (2015) The implications of aquaculture policy and regulation for the development of integrated multi-trophic aquaculture in Europe. Aquaculture 443:16–23. 10.1016/j.aquaculture.2015.03.005

[CR5] Bainbridge JM, Potts T, O’Higgins TG (2011) Rapid policy network mapping: a new method for understanding governance structures for implementation of marine environmental policy. PLoS One 6(10):e26149. 10.1371/journal.pone.0026149 22022545 10.1371/journal.pone.0026149 PMC3193531

[CR6] Bakker K (2014) The business of water: market environmentalism in the water sector. Annu Rev Environ Resour 39(1):469–494. 10.1146/annurev-environ-070312-132730

[CR7] Bannink D, Ossewaarde R (2012) Decentralization: new modes of governance and administrative responsibility. Adm Soc 44(5):595–624. 10.1177/0095399711419096

[CR8] Barbanente A, Grassini L (2022) Fostering transitions in landscape policies: a multi-level perspective. Land Use Policy 112:105869. 10.1016/j.landusepol.2021.105869

[CR9] Bastmeijer K, van Rijswick M, Verschuuren J (2021) Verdroginging Brabant: Een Europees Rechtelijk Perspectief. Tilburg University

[CR10] Beleidstafel Droogte (2019) Nederland beter weerbaar tegen droogte. https://www.rijksoverheid.nl/documenten/rapporten/2019/12/18/eindrapportage-beleidstafel-droogte. Accessed 14 June 2024

[CR11] Berbel J, Esteban E (2019) Droughts as a catalyst for water policy change. Analysis of Spain, Australia (MDB), and California. Glob Environ Change 58:101969. 10.1016/j.gloenvcha.2019.101969

[CR12] Besluit Kwaliteit Leefomgeving (2024) https://wetten.overheid.nl/BWBR0041313/2024-01-01. Accessed 28 May 2024

[CR13] Böcher M (2012) A theoretical framework for explaining the choice of instruments in environmental policy. For Policy Econ 16:14–22. 10.1016/j.forpol.2011.03.012

[CR14] Bodansky D (2016) The legal character of the Paris agreement. Rev Eur Comp Int Environ Law 25(2):142–150. 10.1111/reel.12154

[CR15] Boeuf B, Fritsch O, Martin-Ortega J (2016) Undermining European environmental policy goals? The EU water framework directive and the politics of exemptions. Water 8(9):388. 10.3390/w8090388

[CR16] Borrás S, Edquist C (2013) The choice of innovation policy instruments. Technol Forecast Soc Change 80(8):1513–1522. 10.1016/j.techfore.2013.03.002

[CR17] Bressers H, Bressers N, Kuks S, Larrue C (2016) The governance assessment tool and its use. In: Bressers H, Bressers N, Larrue C (eds) Governance for Drought resilience. Springer International Publishing, pp 45–65. 10.1007/978-3-319-29671-5_3

[CR18] Bressers H, Bressers N, Larrue C (eds) (2016) Governance for drought resilience: land and water drought management in Europe. Springer International Publishing. 10.1007/978-3-319-29671-5

[CR19] Brockhoff RC, Biesbroek R, Van der Bolt B (2022) Drought governance in transition: a case study of the Meuse River Basin in the Netherlands. Water Resour Manage 36(8):2623–2638. 10.1007/s11269-022-03164-7

[CR20] Browne AL, Dury S, De Boer C, La Jeunesse I, Stein U (2016) Governing for drought and water scarcity in the context of flood disaster recovery: the curious case of Somerset, United Kingdom. Governance for Drought Resilience 83–107. 10.1007/978-3-319-29671-5_5

[CR21] Consolidated Version of the Treaty on the Functioning of the European Union (2016) https://data.europa.eu/eli/treaty/tfeu_2016/2024-09-01/. Accessed 6 Oct 2024

[CR22] Copetti D, Erba S (2023) A bibliometric review on the Water Framework Directive twenty years after its birth. Ambio. 10.1007/s13280-023-01918-0 10.1007/s13280-023-01918-0 PMC10692058 37684553

[CR23] Council Directive 92/43/EEC of 21 May 1992 on the conservation of natural habitats and of wild fauna and flora (1992) https://eurlex.europa.eu/legal-content/EN/TXT/?uri=CELEX:01992L0043-20130701. Accessed 7 May 2025

[CR24] Deltaplan Agrarisch Waterbeheer Overijsselse Maatregelen (2023) https://agrarischwaterbeheer.nl/regios/?filterprovincie=overijssel. Accessed 4 June 2024

[CR25] Deltaplan Zoetwater 2022–2027 (2021) https://www.deltaprogramma.nl/documenten/2021/09/21/dp2022-d-deltaplan-zoetwater-2022-2027. Accessed 21 June 2024

[CR26] Directive (EU) 2024/3019 of the European Parliament and of the Council of 27 November 2024 concerning urban wastewater treatment (recast) (Text with EEA relevance) (2024) https://eur-lex.europa.eu/eli/dir/2024/3019/2024-12-12. Accessed 26 Mar 2025

[CR27] Directive 2000/60/EC of the European Parliament and of the Council of 23 October 2000 establishing a framework for Community action in the field of water policy (2000)

[CR28] Directive 2006/118/EC of the European Parliament and of the Council of 12 December 2006 on the protection of groundwater against pollution and deterioration (2006) https://eurlex.europa.eu/legal-content/EN/TXT/?uri=CELEX:02006L0118-20140711. Accessed 7 May 2025

[CR29] Directive 2009/147/EC of the European Parliament and of the Council of 30 November 2009 on the conservation of wild birds (Codified version) (2009) https://eur-lex.europa.eu/legal-content/EN/TXT/?uri=CELEX:02009L0147-20190626. Accessed 7 May 2025

[CR30] Drinkwaterwet (2009) https://wetten.overheid.nl/jci1.3:c:BWBR0026338&z=2024-01-01&g=2024-01-01. Accessed 11 Oct 2024

[CR31] Echeverri A, Furumo PR, Moss S, Figot Kuthy AG, García Aguirre D, et al. (2023) Colombian biodiversity is governed by a rich and diverse policy mix. Nat Ecol Evol 7(3):382–392. 10.1038/s41559-023-01983-4 36747078 10.1038/s41559-023-01983-4

[CR32] Ejelöv E, Nilsson A (2020) Individual factors influencing acceptability for environmental policies: a review and research agenda. Sustainability 12(6):2404. 10.3390/su12062404

[CR33] Fermeglia M (2023) When decentralization strikes back: the example of climate governance in Belgium. Revista d’Estudis Autonòmics i Federals 37:93–126. 10.57645/20.8080.01.4

[CR34] Furusho C, Vidaurre R, La Jeunesse I, Ramos M-H (2016) Cross-cutting perspective freshwater. In: Bressers H, Bressers N, Larrue C (eds) Governance for drought resilience, Springer International Publishing, pp 217–230. 10.1007/978-3-319-29671-5_11

[CR35] Gemeente Enschede (2024) Omgevingsplan gemeente Enschede. https://lokaleregelgeving.overheid.nl/CVDR696198/2. Accessed 2 Apr 2025

[CR36] Gemeente Enschede (2025) Verordening kwaliteit leefomgeving gemeente Enschede 2023. https://lokaleregelgeving.overheid.nl/CVDR696115/3. Accessed 2 Apr 2025

[CR37] Gemeentewet (2025) https://wetten.overheid.nl/jci1.3:c:BWBR0005416&z=2025-02-12&g=2025-02-12. Accessed 11 Jul 2025

[CR38] Gollata JAM, Newig J (2017) Policy implementation through multi-level governance: analysing practical implementation of EU air quality directives in Germany. J Eur Public Policy 24(9):1308–1327. 10.1080/13501763.2017.1314539

[CR39] Guerrin J, Bouleau G, Grelot F (2014) “Functional fit” versus “politics of scale” in the governance of floodplain retention capacity. J Hydrol 519:2405–2414. 10.1016/j.jhydrol.2014.08.024

[CR40] Gunningham N, Sinclair D (1999) Regulatory pluralism: designing policy mixes for environmental protection. Law Pol 21(1):49–76. 10.1111/1467-9930.00065

[CR41] Havekes H, Lavrysen L, Hildering A (2024) Waterbeheer in de Lage Landen. Koninklijk Nederlands Waternetwerk. https://www.waternetwerk.nl/images/knw2/2407-Waterbeheer-in-de-Lage-Landen-Boek.pdf. Accessed 21 Mar 2025

[CR42] Hervás-Gámez C, Delgado-Ramos F (2019) Drought management planning policy: from Europe to Spain. Sustainability 11(7):1862. 10.3390/su11071862

[CR43] Heyl K, Döring T, Garske B, Stubenrauch J, Ekardt F (2020) The common agricultural policy beyond 2020: a critical review in light of global environmental goals. Rev Eur Comp Int Environ Law. 10.1111/reel.12351

[CR44] Heyvaert V (2018) Transnational environmental regulation and governance: purpose, strategies and principles (1st ed.). Cambridge University Press. 10.1017/9781108235099

[CR45] Hölscher K, Frantzeskaki N, Loorbach D (2019) Steering transformations under climate change: capacities for transformative climate governance and the case of Rotterdam, the Netherlands. Reg Environ Change 19(3):791–805. 10.1007/s10113-018-1329-3

[CR46] Huang J, Hartemink AE (2020) Soil and environmental issues in sandy soils. Earth Sci Rev 208:103295. 10.1016/j.earscirev.2020.103295

[CR47] Hurlimann AC, March AP (2012) The role of spatial planning in adapting to climate change. Wires Clim Change 3(5):477–488. 10.1002/wcc.183

[CR48] Jain P, Barber QE, Taylor SW, Whitman E, Castellanos Acuna D, et al. (2024) Drivers and impacts of the record-breaking 2023 wildfire season in Canada. Nat Commun 15(1):6764. 10.1038/s41467-024-51154-7 39164286 10.1038/s41467-024-51154-7 PMC11335882

[CR49] Jancewicz KA (2024) Does the European Union need a ‘drought directive’? a legal perspective. Teisė 130:55–65. 10.15388/Teise.2024.130.5

[CR50] Jiang J, Zhou T (2023) Agricultural drought over water-scarce Central Asia aggravated by internal climate variability. Nat Geosci 16(2):154–161. 10.1038/s41561-022-01111-0

[CR51] Kastelein R (2024) Role of local governments in EU member states’ climate policy and legislation. npj Clim Action 3:92. 10.1038/s44168-024-00177-3

[CR52] Keessen AM, Van Rijswick HFMW (2012) Adaptation to climate change in European water law and policy. Utrecht Law Rev 8(3):38. 10.18352/ulr.204

[CR53] Kraehnert K, Osberghaus D, Hott C, Habtemariam LT, Wätzold F, et al. (2021) Insurance against extreme weather events: an overview. Rev Econ 72(2):71–95. 10.1515/roe-2021-0024

[CR54] Lake PS (2003) Ecological effects of perturbation by drought in flowing waters. Freshw Biol 48(7):1161–1172. 10.1046/j.1365-2427.2003.01086.x

[CR55] Leshinsky R, Legacy C (2016) Instruments of planning: tensions and challenges for more equitable and sustainable cities. Routledge

[CR56] Lloyd-Hughes B (2014) The impracticality of a universal drought definition. Theoret Appl Climatol 117(3–4):607–611. 10.1007/s00704-013-1025-7

[CR57] Nationaal Deltaprogramma 2024 (2023) https://www.deltaprogramma.nl/site/binaries/sitecontent/collections/documents/2023/09/19/dp024/DP2024.pdf. Accessed 1 Sept 2024

[CR58] Nationaal Water Programma 2022–2027 (2022) https://www.rijksoverheid.nl/documenten/rapporten/2022/03/18/bijlage-nationaalwater-programma-2022-2027. Accessed 31 Aug 2024

[CR59] Nationale Adaptatie Strategie (2016) https://klimaatadaptatienederland.nl/beleid/nationale-aanpak/nas/. Accessed 19 June 2024

[CR60] Nationale Omgevingsvisie (NOVI) (2020) https://klimaatadaptatienederland.nl/publish/pages/182693/nationale_omgevingsvisie_novi.pdf. Accessed 31 Aug 2024

[CR61] Ohlhorst D (2015) Germany’s energy transition policy between national targets and decentralized responsibilities. J Integr Environ Sci 12(4):303–322. 10.1080/1943815X.2015.1125373

[CR62] Omgevingswet (2024) https://wetten.overheid.nl/BWBR0037885/2024-01-01. Accessed 8 May 2025

[CR63] Pahl-Wostl C, Holtz G, Kastens B, Knieper C (2010) Analyzing complex water governance regimes: the management and transition framework. Environ Sci Policy 13(7):571–581. 10.1016/j.envsci.2010.08.006

[CR64] Projectteam Droogte Zandgronden Nederland (2021) Droogte in zandgebieden van Zuid-, Midden- en Oost-Nederland. https://klimaatadaptatienederland.nl/@253692/droogte-zandgebieden-van-zuid-midden-en/. Accessed 14 Oct 2024

[CR65] Provincie Brabant (2024) Verbreding grondwaterheffing: Meer bedrijven gaan betalen voor grondwatergebruik. https://www.brabant.nl/actueel/nieuws/verbreding-grondwaterheffingbedrijven/. Accessed on 28 Mar 2025

[CR66] Provincie Noord-Brabant (2024) Verordening grondwaterheffing Noord-Brabant 2023. https://lokaleregelgeving.overheid.nl/CVDR680523/1. Accessed 28 Mar 2025

[CR67] Provincie Overijssel (2022) Fundament voor de Nieuwe Omgevingsvisie. https://www.overijssel.nl/media/rqvnwtv2/fundamentomgevingsvisie-gec.pdf. Accessed 21 June 2024

[CR68] Provincie Overijssel (2024b) Natuurbeheerplan 2025 Provincie Overijssel. https://lokaleregelgeving.overheid.nl/CVDR724286/1. Accessed 27 May 2025

[CR69] Provincie Overijssel (2024e) Belastingverordening Overijssel. https://lokaleregelgeving.overheid.nl/CVDR707383/. Accessed 31 Aug 2024

[CR70] Provincie Overijssel (2024f) Omgevingsverordening Overijssel. https://lokaleregelgeving.overheid.nl/CVDR706717/1. Accessed 14 Oct 2024

[CR71] Provincie Overijssel (2024g) Concept Nieuwe Omgevingsvisie Overijssel. https://overijssel.notubiz.nl/document/14331435/1. Accessed 22 Aug 2024

[CR72] Provincie Overijssel (2024c) Subsidieregeling kwaliteitsimpuls natuur en landschap Overijssel 2024. https://lokaleregelgeving.overheid.nl/CVDR704770/1. Accessed 1 Oct 2024

[CR73] Provincie Overijssel (2024d) Subsidieregeling natuur- en landschapsbeheer Overijssel 2024. https://lokaleregelgeving.overheid.nl/CVDR704720/4. Accessed 2 October 2024

[CR74] Provincie Overijssel (2024a) Uitvoeringsbesluit subsidies Overijssel 2022. https://lokaleregelgeving.overheid.nl/CVDR679264/20. Accessed 11 Oct 2024

[CR75] Provinciewet (2025) https://wetten.overheid.nl/jci1.3:c:BWBR0005645&z=2025-02-12&g=2025-02-12. Accessed 11 Jul 2025

[CR76] Regulation (EU) 2020/741 of the European Parliament and of the Council of 25 May 2020 on minimum requirements for water reuse (2020) https://eur-lex.europa.eu/eli/reg/2020/741/oj/eng. Accessed 2 Apr 2025

[CR77] Regulation (EU) 2021/1119 of the European Parliament and of the Council of 30 June 2021 establishing the framework for achieving climate neutrality and amending Regulations (EC) No 401/2009 and (EU) 2018/1999 (‘European Climate Law’) (2021) https://eur-lex.europa.eu/legal-content/EN/TXT/?uri=CELEX:32021R1119. Accessed 7 May 2025

[CR78] Regulation (EU) 2021/2115 establishing rules on support for strategic plans to be drawn up by Member States under the common agricultural policy (CAP Strategic Plans) and financed by the European Agricultural Guarantee Fund (EAGF) and by the European Agricultural Fund for Rural Development (EAFRD) and repealing Regulations (EU) No 1305/2013 and (EU) No 1307/2013 (2024) https://eur-lex.europa.eu/legal-content/EN/TXT/?uri=CELEX:02021R2115-20240525. Accessed 3 Apr 2025

[CR79] Regulation (EU) 2024/1991 of the European Parliament and of the Council of 24 June 2024 on nature restoration and amending Regulation (EU) 2022/869 (2024) https://eur-lex.europa.eu/legal-content/EN/TXT/?uri=CELEX:32024R1991. Accessed 13 Mar 2025

[CR80] Reich P, Lake PS (2015) Extreme hydrological events and the ecological restoration of flowing waters. Freshw Biol 60(12):2639–2652. 10.1111/fwb.12508

[CR81] Rey D, Pérez-Blanco CD, Escriva-Bou A, Girard C, Veldkamp TIE (2019) Role of economic instruments in water allocation reform: lessons from Europe. Int J Water Resour Dev 35(2):206–239. 10.1080/07900627.2017.1422702

[CR82] Rogge KS, Reichardt K (2016) Policy mixes for sustainability transitions: an extended concept and framework for analysis. Res Policy 45(8):1620–1635. 10.1016/j.respol.2016.04.004

[CR83] Ruiz Estrada MA (2011) Policy modeling: definition, classification and evaluation. J Policy Model 33(4):523–536. 10.1016/j.jpolmod.2011.02.003

[CR84] Sari R, Soytas U, Kanoglu-Ozkan DG, Sivrikaya A (2023) Improving the climate resilience of European cities via socially acceptable nature-based solutions. Npj Urban Sustain 3(1):9. 10.1038/s42949-023-00090-4

[CR85] Sarremejane R, Messager ML, Datry T (2022) Drought in intermittent river and ephemeral stream networks. Ecohydrol 15(5):e2390. 10.1002/eco.2390

[CR86] Schuerhoff M, Weikard H-P, Zetland D (2013) The life and death of Dutch groundwater tax. Water Policy 15(6):1064–1077. 10.2166/wp.2013.112

[CR87] Silvis HJ, Voskuilen MJ, Kuiper M, Van Essen E (2016) Grondsoort en grondprijs. 10.18174/394962

[CR88] Spinoni J, Naumann G, Carrao H, Barbosa P, Vogt J (2014) World drought frequency, duration, and severity for 1951–2010: world drought climatologies for 1951–2010. Int J Climatol 34(8):2792–2804. 10.1002/joc.3875

[CR89] Starke JR, Van Rijswick HFMW (2021) Exemptions of the EU water framework directive deterioration ban: comparing implementation approaches in Lower Saxony and the Netherlands. Sustainability 13(2):930. 10.3390/su13020930

[CR90] Stead D (2021) Conceptualizing the policy tools of spatial planning. J Plan Lit 36(3):297–311. 10.1177/0885412221992283

[CR91] Stein U, Özerol G, Troeltzsch J, Landgrebe R, Szendrenyi A, Vidaurre R (2016) European drought and water scarcity policies. In: H Bressers, N Bressers & C Larrue (eds) Governance for Drought Resilience: Land and Water Drought Management in Europe. Springer International Publishing, pp 17–43. 10.1007/978-3-319-29671-5_2

[CR92] Sweet SK, Wolfe DW, DeGaetano A, Benner R (2017) Anatomy of the 2016 drought in the Northeastern United States: implications for agriculture and water resources in humid climates. Agric for Meteorol 247:571–581. 10.1016/j.agrformet.2017.08.024

[CR93] Taylor C, Pollard S, Rocks S, Angus A (2012) Selecting policy instruments for better environmental regulation: a critique and future research agenda. Environ Policy Gov 22(4):268–292. 10.1002/eet.1584

[CR94] The Netherlands National Strategic Plan CAP 2023–2027 (2023) https://www.netwerkplatteland.nl/het-gemeenschappelijklandbouwbeleid-2023-2027/documenten/publicaties/2022/11/02/programmadocument-nsp-glb-2023-2027-versie-1.3. Accessed 8 Jul 2024

[CR95] Thieme M, Birnie-Gauvin K, Opperman JJ, Franklin PA, Richter H et al (2024) Measures to safeguard and restore river connectivity. Environ Rev 32(3):366–386. 10.1139/er-2023-0019

[CR96] Thomann E, Sager F (2017) Moving beyond legal compliance: innovative approaches to EU multilevel implementation. J Eur Public Policy 24(9):1253–1268. 10.1080/13501763.2017.1314541

[CR97] Turnheim B, Berkhout F, Geels F, Hof A, McMeekin A, et al. (2015) Evaluating sustainability transitions pathways: bridging analytical approaches to address governance challenges. Glob Environ Chang 35:239–253. 10.1016/j.gloenvcha.2015.08.010

[CR98] van der Horst D (2006) Spatial cost–benefit thinking in multi-functional forestry; towards a framework for spatial targeting of policy interventions. Ecol Econ 59(1):171–180. 10.1016/j.ecolecon.2005.10.005

[CR99] Van Der Jagt A, Tozer L, Toxopeus H, Runhaar H (2023) Policy mixes for mainstreaming urban nature-based solutions: an analysis of six European countries and the European Union. Environ Sci Policy 139:51–61. 10.1016/j.envsci.2022.10.011

[CR100] van Dijk T (2005) The dangers of transplanting planning instruments: the case of land fragmentation in Central Europe. Eur J Spat Dev 3(3):1–39. 10.5281/zenodo.5127812

[CR101] van Houtert L (2024, August 30) Wie grondwater oppompt, moet vanaf 1 januari betalen—Maar hoeveel is nog onduidelijk. Brabants Dagblad. https://www.bd.nl/eindhoven/wie-grondwater-oppompt-moet-vanaf-1-januari-betalen-maar-hoeveel-is-nog-onduidelijk~a9e8fd65/

[CR102] van Hussen K, van de Velde I, Läkamp R, van der Kooij S, Hekman A (2019) Economische schade door droogte in 2018. In: Rotterdam, NL, Ecorys. https://zoek.officielebekendmakingen.nl/blg-916915.pdf. Accessed 18 Sept 2024

[CR103] Verordening kwaliteit leefomgeving gemeente Enschede 2023 (2025)

[CR104] Waterschap Vechtstromen (2020) Watervisie 2050 Waterschap Vechtstromen. https://vechtstromen.bestuurlijkeinformatie.nl/Agenda/Document/07f3561a-6365-4430-9bcc-712a6f83d9b8?documentId=da0b3c8c-734c-4e0c-837f-122fabb6cc97&agendaItemId=fc921d3f-a215-4123-a111-cd21dceb5168. Accessed 31 Aug 2024

[CR105] Waterschap Vechtstromen (2021) Waterbeheerprogramma 2022–2027 Waterschap Vechtstromen. https://vechtstromen.bestuurlijkeinformatie.nl/Agenda/Document/dd2aaa8a-eae3-4a00-bc99-7542955a6eff?documentId=0967098fe41a-4982-a0df-e670d3cb77d1&agendaItemId=4dacd9de-ff3d-408f-93a8-555db1076c89. Accessed 15 Aug 2024

[CR106] Waterschap Vechtstromen (2024) Waterschapsverordening Waterschap Vechtstromen. https://lokaleregelgeving.overheid.nl/CVDR705379/1. Accessed 19 Sept 2024

[CR107] Waterwet (2024) https://wetten.overheid.nl/BWBR0025458/2024-01-01. Accessed 13 May 2025

[CR108] Wet Revitalisering Generiek Toezicht (2012) https://wetten.overheid.nl/BWBR0031628/2013-01-01. Accessed 11 Jul 2025

[CR109] Wuijts S, Van Rijswick HF, Driessen PP, Runhaar HA (2023) Moving forward to a the ambitions of the European water framework directive: lessons learned from the Netherlands. J Environ Manage 333:117424. 10.1016/j.jenvman.2023.117424 36764178 10.1016/j.jenvman.2023.117424

