# Supplementary material for: Catching up with drought: law and policy responses in the Netherlands
Source: Reg Environ Change. 2025 Aug 20;25(3):112. doi: 10.1007/s10113-025-02449-y (PMC12367876; doi:10.1007/s10113-025-02449-y)
Supplement: Supplementary file 1 — Supplementary file1 (DOCX 65 KB) [file 10113_2025_2449_MOESM1_ESM.docx]

**Supporting Information**

*Title:*

**Catching up with drought: law and policy responses in the Netherlands.**

*Journal:*

**Regional Environmental Change**

*Author name(s):*

Max Frederik Wicher Augustijn^1,2^ *(corresponding author)*

Hollandseweg 1, 6706 KN Wageningen, The Netherlands

[max.augustijn@wur.nl](mailto:max.augustijn@wur.nl)

+31 6 290 99 300

Edwin Alblas^1^

Andries Richter^2^

*Affiliation(s):*

^1^ Wageningen University & Research, Law Group

^2^ Wageningen University & Research, Environmental Economics and Natural Resources Group

# Appendix A: Geospatial data sources

| **Layer** | **Figure** | **Source** | **Data Processing** |
| --- | --- | --- | --- |
| Precipitation deficiency | Figure 1 | Royal Netherlands Meteorological Institute (KNMI). (2023). Archief neerslagoverschot [PNG]. Retrieved from https://www.knmi.nl/nederland-nu/klimatologie/geografische-overzichten/archief-neerslagoverschot | Created an average of the map layers of three years by tracing, overlaying and averaging the raster values. |
| Sandy soil types | Figure 1 | Ministerie van Binnenlandse Zaken en Koninkrijksrelaties. (2023). Basisregistratie Ondergrond (BRO) [GPKG]. Retrieved from https://www.pdok.nl/basisregistratie-ondergrond | A filter function was used on the soil types map to select only sandy soil types. |
| European Union border | Figure 2 | World Food Programme. 2019. World Administrative Boundaries - Countries and Territories [Geojson]. Retrieved from https://public.opendatasoft.com/explore/dataset/world-administrative-boundaries/export/ | Filtered European Union Member States. |
| National border | Figure 2 | Esri Nederland. (2021). Landsgrens actueel [Geopackage]. Retrieved from https://www.arcgis.com/home/item.html?id=66ad4073c8284379b676cddfe1f0350d | – |
| Twente border | Figure 2 | Esri Nederland. (2020). Regionale Energiestrategie (RES) regio's. Retrieved from https://hub.arcgis.com/datasets/6d91187a2f9f4bc589d2c6fb5699d7c0_0/explore?location=52.120839%2C5.248442%2C8.07 | – |
| Provincial borders | Figures 2-6 | Kadaster. (2021). Bestuurlijke Grenzen [Geopackage]. Retrieved from https://www.pdok.nl/introductie/-/article/bestuurlijke-grenzen | – |
| Water authority borders | Figures 2-6 | Esri Nederland, Kadaster & Unie van Waterschappen. (2023). Waterschaps Grenzen [Geojson] Retrieved from https://hub.arcgis.com/datasets/7adde9c6ad2d454780a725af3c42f3f2_0/explore?location=52.093096%2C5.248626%2C8.11 | – |
| Groundwater extraction areas; Groundwater protection areas; Infiltration areas; Soil drilling prohibited zones | Figure 4 | Ministerie van Infrastructuur en Waterstaat. (2023). Grondwaterbeschermingskaart rondom bronnen voor drinkwater [Shapefile]. Retrieved from https://www.atlasleefomgeving.nl/grondwaterbeschermingskaart-rondom-bronnen-voor-drinkwater | – |
| National Waters | Figure 4 | Informatiehuis Water. (2024). KRW Oppervlaktewaterlichamen [GML] Retrieved from https://www.pdok.nl/introductie/-/article/krw-oppervlaktewaterlichamen-inspire-geharmoniseerd- | – |
| Twente Canals | Figure 4 | Informatiehuis Water. (2024). KRW Oppervlaktewaterlichamen [GML] Retrieved from https://www.pdok.nl/introductie/-/article/krw-oppervlaktewaterlichamen-inspire-geharmoniseerd- | Filtered the Twente canals from the National Waters layers, following the National Water Program (2022). |
| Regional water bodies managed by water authorities under WFD | Figure 4 | Overijssel. (2023). Kaderrichtlijn water (lijnen) [Shapefile]. Retrieved from https://www.geoportaaloverijssel.nl/metadata/dataset/3d38d772-d0ea-4654-8b87-f00b9dc75298 | The dataset also contains WFD regional water bodies in the Vechtstromen region situated in the province of Drenthe. No data processing was required. |
| Supplementary Strategic Reserves | Figure 4 | Ministerie van Infrastructuur en Waterstaat. (2023). Grondwaterbeschermingskaart rondom bronnen voor drinkwater [Shapefile]. Retrieved from https://www.atlasleefomgeving.nl/grondwaterbeschermingskaart-rondom-bronnen-voor-drinkwater | Supplementary Strategic Reserves overlap with ground water protection areas, infiltration areas and soil drilling prohibited zones, therefore this layer was computed by selecting the areas in question. |
| Areas for water retention | Figure 4 | Overijssel. (2023). Waterbergingsgebieden [GML]. Retrieved from https://zoek.officielebekendmakingen.nl/dc-2023-4509/1/html | – |
| Natura 2000 | Figure 5 | RVO. (2024). Natura 2000-gebieden [Geojson]. Retrieved from https://nationaalgeoregister.nl/geonetwork/srv/dut/catalog.search#/metadata/8829e5dd-c861-4639-a6c8-fdbb6e3440d2 | – |
| Nature Network Netherlands | Figure 5 | BIJ12. (2024). Natuurnetwerk Nederland (NNN). Retrieved from https://www.pdok.nl/atom-downloadservices/-/article/natuurnetwerk-nederland-nnn-#722718366f8d7f76d74fc1fc6421fae7 | – |
| Pine Forests | Figure 5 | Kadaster. (2023). Basisregistratie Topografie (BRT) [Geopackage]. Retrieved from https://www.pdok.nl/introductie/-/article/basisregistratie-topografie-brt-topnl | A filter function was used to select only pine forests. |
| Entrepreneurship with water zones outside of Nature Network Netherlands | Figure 5 | Overijssel. (2024). Begrenzing NNN en Zone Ondernemen met Natuur en Water [Shapefile]. Retrieved from https://www.geoportaaloverijssel.nl/metadata/dataset/e32ecb1c-a023-4db7-b2d0-f889ee6fac46 | The layer is arranged underneath the Nature Network Netherlands layer, hereby effectively showing only the areas outside of Nature Network Netherlands. |
| Current Nature and Landscape Management Zones outside of Nature Network Netherlands | Figure 5 | Overijssel. (2024). Subsidiekaart SNLn (Openstellingsbesluit SNL 2025) [Shapefile]. Retrieved from https://www.geoportaaloverijssel.nl/metadata/dataset/323c3f6d-1876-44c5-b8e2-6254467b6bc5 | The layer is arranged underneath the Nature Network Netherlands layer, hereby effectively showing only the areas outside of Nature Network Netherlands. |
| Agricultural land use | Figure 5 | Ministerie van Economische Zaken en Klimaat. (2022). Basisregistratie Gewaspercelen (BRP) [GPKG]. Retrieved from https://service.pdok.nl/rvo/brpgewaspercelen/atom/v1_0/basisregistratie_gewaspercelen_brp.xml | Land use was recategorized into ‘grassland’, ‘corn’, ‘fruits and vegetables’, and so forth. |
| Base map | Figure 5&6 | Kadaster. (2023). Basisregistratie Topografie (BRT) [Geopackage]. Retrieved from https://www.pdok.nl/introductie/-/article/basisregistratie-topografie-brt-topnl | Used the TOP10NL dataset. |
| Stream Valleys; Sand ridges; Moraines | Figure 6 | Ministerie van Binnenlandse Zaken en Koninkrijksrelaties (Rijk). (2023). Basisregistratie Ondergrond (BRO) [GPKG]. Retrieved from https://www.pdok.nl/basisregistratie-ondergrond | A filter function on the geomorphological map was used to select only stream valleys, sand ridges and moraines. |
| Areas without access to non-local water intake | Figure 6 | Projectteam Droogte Zandgronden Nederland. (2021). Droogte in zandgebieden van Zuid-, Midden- en Oost-Nederland. | Traced from the map presented in Projectteam Droogte Zandgronden Nederland (2021). |
| Altitude (mini-)map | Figure 6 | Algemeen Hoogtebestand Nederland. (2023). Actueel Hoogtebestand Nederland (AHN4) [WCS]. Rijkswaterstaat. Retrieved from https://www.nationaalgeoregister.nl/geonetwork/srv/api/records/bfcc588f-9393-4c70-b989-d9e92ac2f493 | Used the DTM 0.5m layer |
| Water extraction from surface and ground water for irrigation | Figure 6 | Rijksinstituut voor Volksgezondheid en Milieu. (2024). Irrigatiewater - Locatie beregeningsonttrekkingen uit grondwater en oppervlaktewater [Shapefile]. Retrieved from  https://data.overheid.nl/dataset/25322-irrigatiewater---locatie-beregeningsonttrekkingen-uit-grondwater-en-oppervlaktewater#panel-resources | – |
| Water extraction from surface water for drinking water production | Figure 6 | Rijksinstituut voor Volksgezondheid en Milieu. (2024). DANK - Gebruik en behoefte aan zoetwater voor diverse functies & locaties industriewateronttrekking [Shapefile]. Retrieved from https://data.overheid.nl/en/dataset/33057-dank--gebruik-en-behoefte-aan-zoetwater-voor-diverse-functies--locaties-drinkwateronttrekkinge | – |
| Water extraction from surface water for industry | Figure 6 | Rijksinstituut voor Volksgezondheid en Milieu. (2024). DANK - Gebruik en behoefte aan zoetwater voor diverse functies & locaties industriewateronttrekking [Shapefile]. Retrieved from https://data.overheid.nl/en/dataset/33059-dank--gebruik-en-behoefte-aan-zoetwater-voor-diverse-functies--locaties-industriewateronttrekk | – |
| Water extraction from groundwater for drinking water and industry in the province of Overijssel | Figure 6 | Rekenkamer Oost-Nederland. (2022). Als elke druppel telt. Retrieved from https://rekenkameroost.nl/als-elke-druppel-telt/ | Traced the accompanying PNG file. |
| Water extraction from groundwater for drinking water in the province of Drenthe | Figure 6 | Drenthe. (2021). Regionaal Waterprogramma Drenthe 2022-2027. Retrieved from https://lokaleregelgeving.overheid.nl/CVDR667652 | Traced the accompanying PNG file. Data on Water extraction from groundwater for industry was not available for the province of Drenthe. |
|  |  |  |  |

# Appendix B: Translations and abbreviations

| **Dutch** | **English** | **Abbreviation** |
| --- | --- | --- |
| Belastingverordening | Tax Regulation |  |
| Beleidstafel Droogte | Drought Policy Platform |  |
| Besluit Kwaliteit Leefomgeving | Decision on the Quality of the Living Environment |  |
| Catalogus Gebiedskenmerken | Catalogue of Area Characteristics |  |
| Deltaplan Agrarisch Waterbeheer | Delta Plan Agricultural Water Management |  |
| Deltaplan Zoetwater | Delta Plan Freshwater |  |
| Drinkwaterwet | Drinking Water Act |  |
| Gemeenschappelijk Landbouw Beleid | Common Agricultural Policy | CAP |
| Gemeentewet | Municipal Government Act |  |
| Interbestuurlijk toezicht | Intergovernmental Oversight |  |
| Kaderrichtlijn Water | Water Framework Directive | WFD |
| Klimaatwet | Climate Law |  |
| Nationaal Deltaprogramma | National Delta Program |  |
| Nationaal Strategisch Plan (CAP) | National Strategic Plan (CAP) |  |
| Nationaal Uitvoeringsprogramma Klimaatadaptatie | National Implementation Program Climate Adaptation |  |
| Nationaal Water Programma | National Water Program |  |
| Nationale Adaptatie Strategie | National Adaptation Plan |  |
| Nationale Omgevingsvisie | National Environment and Planning Strategy |  |
| Omgevingswet | Environment and Planning Act |  |
| Omgevingsplan (gemeente) | Municipal Environmental Plan |  |
| Omgevingsvergunning | Environmental Permit |  |
| Provinciaal Natuurbeheerplan | Provincial Nature Management Plan |  |
| Provinciale Belastingsverordening | Provincial Tax Regulation |  |
| Provinciale Omgevingsverordening | Provincial Environment and Planning Regulation |  |
| Provinciale Omgevingsvisie | Provincial Environment and Planning Strategy |  |
| Provinciewet | Provincial Government Act |  |
| Stroomgebiedbeheerplan | River Basin Management Plan |  |
| Subsidieregeling Kwaliteitsimpuls Natuur en Landschap | Quality Impulse Nature and Landscape subsidy scheme | SKNL |
| Subsidieregeling Natuur en Landschapsbeheer | Nature and Landscape Management subsidy scheme | SRNL, ANLb |
| Uitvoeringsbesluit Subsidies | Executive Decree Subsidies |  |
| Vogel-en Habitatrichtlijnen | Birds and Habitats Directives |  |
| Verordening Grondwaterheffing | Groundwater Tax Regulation |  |
| Verordening Kwaliteit Leefomgeving (gemeente) | Municipal Regulation on the Quality of the Living Environment |  |
| Waterbeheer Programma | Water Management Program |  |
| Waterschapsverordening | Water Authority Regulation |  |
| Watervisie | Water Strategy |  |
| Waterwet | Water Act |  |
| Wet Revitalisering Generiek Toezicht | Law on Revitalizing Generic Supervision |  |
|  |  |  |

# Appendix C: Extended Descriptions of Drought Policies

## European Union Water Framework Directive

The Water Framework Directive (Directive 2000/60/EC, 2000, 'WFD') is directly concerned with water governance, aiming to “establish a framework for the protection of inland surface waters, transitional waters, coastal waters, and groundwater”, which includes mitigating floods and droughts (Bastmeijer et al., 2021; Copetti & Erba, 2023; article 1(e) WFD). Drought is addressed with command-and-control instruments presented in six articles that concern the roles of Member State governments and the contents of the WFD mandated River Basin Management Plans and Programmes of Measures. First, article 4(b) mandates the protection of groundwater by balancing abstraction and recharge in Member States, to preserve water resources used for drinking water and irrigation, and to preserve the upward ground water pressure by which plants naturally uptake water. Second, article 6 mandates the establishment of surface and ground water protection areas by Member States to conserve water-dependent habitats and species, bathing water and drinking water supplies. Third, article 7 mandates the identification of water bodies used for water extraction by Member States and requires Member States to ensure the necessary protection with the aim of avoiding deterioration in their quality. Fourth, article 8 contains provisions on the establishment of monitoring programs by Member States covering the chemical and quantitative status of groundwater bodies and of the chemical, quantitative and ecological status of groundwater bodies. Fifth, article 9 mandates adequate water pricing to incentivize efficient water use, thus addressing overextraction of water resources. Finally, article 11 concerns the inclusion of “supplementary” drought-specific measures in Member States’ Programmes of Measures “where necessary”, such as promoting low-water requiring crops in drought-affected areas.

## European Union Common Agricultural Policy

The Common Agricultural Policy (‘CAP’) is historically aimed at ensuring a stable food supply and safeguarding farmer incomes (article 39 TFEU, 2016). Recent reforms, however, have integrated climate change mitigation, adaptation, and sustainable resource management objectives (Heyl et al., 2020; i.e., articles 5, 6 and 105 of Regulation (EU) 2021/2115, 2024). Although the CAP does not explicitly mention drought outside of the preamble, certain conditionalities for direct income support, eco-scheme subsidies, sector-specific direct payments and European Agricultural Fund for Rural Development payments can be considered to address drought implicitly upon consulting literature. As elaborated upon in the next paragraphs, these provisions are predominantly economic instruments, although they also incorporate command-and-control elements by requiring compliance with specific environmental standards.

Articles 12 and 13 of the CAP Strategic Plan Regulation (Regulation (EU) 2021/2115, 2024) concern the conditionalities (i.e., mandatory requirements) for farmers to receive direct hectare-based income support under the first pillar of the CAP. Although none of the measures explicitly addresses drought, certain conditionalities do address water quantity indirectly (p. 43 in Lampkin et al., 2020): (1) tillage management reducing the risk of soil degradation (GAEC 5); (2) soil cover in the most sensitive period(s) (GAEC 6); (3) crop rotation on arable land (GAEC 7), and; (4) compliance to mandatory requirements from the WFD (article 11(3e)) regarding the authorization of water abstractions (SMR 1). Additionally, conditionalities addressing the maintenance of soil organic matter can be considered to relate indirectly to drought, given that the maintenance of soil organic matter is essential for the retention of water in soils (Lal, 2020). In terms of soil organic matter, GAEC’s 1, 2 and 3 require the maintenance of permanent grassland, the protection of wetlands and peatlands, and a ban on the burning of arable stubble, respectively.

Provisions on the inclusion of voluntary eco-scheme subsidies in Member States’ National Strategic Plans are outlined in article 31 of Regulation (EU) 2021/2115. Simply put, eco-schemes are voluntary direct hectare-based payments that are granted to farmers for sustainable agricultural practices in addition to the conditional direct income support. Article 31(4) pertains that “each eco-scheme shall in principle cover at least two of the following areas of actions for the climate, the environment, animal welfare and combatting antimicrobial resistance”, which includes “protection or improvement of water quality and reduction of pressure on water resources”. Given that eco-schemes are selected by Member States and presented in the CAP National Strategic Plans, the eco-schemes are further discussed in the section on the national layer.

Next, articles 46, 47 and 58 of Regulation (EU) 2021/2115 contain provisions on direct payments for interventions in specifically the fruit, vegetables, hops, olive and wine sectors that can be included in Member States’ National Strategic Plans. These articles state that the objectives of the interventions should relate to physical climate change adaptation interventions, advisory services for sustainable agricultural practices, insurance against adverse climatic events and “sound water management practices”.

The final articles in Regulation (EU) 2021/2115 of relevance to drought governance, are those pertaining to payments from the European Agricultural Fund for Rural Development, i.e., the second pillar of the CAP. Although implicitly at times, the CAP’s second pillar provides financial support for seven measures that relate to drought: (1) climate adaptation related management commitments that go beyond the conditionalities (article 70); (2) farmers in areas with natural and area-specific constraints, such as those impairing a sustainable management of water (articles 6(1) and 71); (3) area-specific disadvantages resulting from mandatory requirements of the WFD and the Birds and Habitats Directives (article 72); (4) investments in the restoration and prevention of damages due to adverse climatic events (article 73); (5) irrigation, when an environmental impact analysis shows that there will be no significant negative environmental impact (article 74); (6) risk management and income stabilization tools, including contributions to premiums for insurance schemes safeguarding incomes as a consequence of adverse climatic events (article 76), and; (7) knowledge exchange specifically targeting the protection of nature, environment and climate (article 78).

## European Union Nature Restoration Regulation

The Nature Restoration Regulation (Regulation (EU) 2024/1991, 2024) aims to restore ecosystems to ensure the recovery of biodiverse and resilient nature in the European Union and, in doing so, aims to contribute to the European Union’s climate change mitigation and climate change adaptation objectives. The Nature Restoration Regulation touches upon drought adaptation in several ways. Articles 4 and 9 pertain requirements for Member State governments to enhance the quality, quantity and connectivity of freshwater ecosystems and to restore the natural connectivity of rivers, respectively, which may imply some degree of enhancement of drought resilience. More specifically, in line with the Birds and Habitats Directives, the restoration of freshwater ecosystems often implies an improvement of hydrological conditions within and around natural areas, as can be further substantiated by the measures listed in the annexes of the Nature Restoration Regulation. Furthermore, restoring the connectivity of freshwater ecosystems and rivers can also contribute to enhancing drought resilience, as this enhances the flow of water during droughts that can be used by natural vegetation and for irrigation, enhances groundwater recharge, and mitigates biodiversity decline by reducing the accumulation of pollution in water bodies during drought and by allowing aquatic species to migrate, among others (Lake, 2003; Reich & Lake, 2015; Sarremejane et al., 2022; Thieme et al., 2024). However, concerns can be raised over the exemptions listed in article 4(14 and 15), which allow for derogations by Member States in improving habitat quality in natural areas outside of Natura 2000 if the deterioration is the consequence of natural disasters, climate change or the situation of a project of overriding public interest in the area. Article 11 and 13 can furthermore be interpreted as potentially contributing to drought resilience as these articles pertain the restoration of agricultural ecosystems and reforestation, respectively, as they both mention climate change adaptation as an objective. Member States are mandated to develop National Restoration Plans (article 14) to outline measures to restore habitats, yet due to the fact that the regulation only recently entered into force, the Dutch National Restoration Plan is not yet published.

## European Union Urban Wastewater Treatment Directive

The Urban Wastewater Treatment Directive (Directive (EU) 2024/3019, 2024) sets the legal framework for the collection, treatment and discharge of urban wastewater and the discharge of biodegradable wastewater from certain industrial sectors. Most concretely addressing drought, is article 15(1), which states that “Member States shall systematically promote the reuse of treated wastewater from all urban wastewater treatment plants where appropriate, especially in water-stressed areas, and for all appropriate purposes”. Furthermore, urban wastewater treatment plants must be built taking into account vulnerability to climate change (article 13). The strategy to implement water reuse are to be featured in Integrated Urban Wastewater Management Plans, which Member States have to establish under the directive for drainage areas of sizable agglomerations within their territory (article 5, 18 and annex V). These integrated urban wastewater management plans are to be established by 2033 for agglomerations of 100,000 population equivalents and above, and 2045 for agglomerations of 10,000 population equivalents and above.

## The Netherlands Environment and Planning Act

The Environment and Planning Act (*Omgevingswet*, 2024) addresses drought on several instances with command-and-control instruments. First, article 2.42 requires national and regional layers to include a hierarchy in societal and ecological necessities in their regulations that is decisive in the allocation of surface water during scarcity, and that may be applied to ground water as well. Second, the Environment and Planning Act contains provisions on water extraction activities, mandating that Provincial Environment Regulations, Municipal Environmental Plans and Water Authority Management Plans must include provisions on water extraction activities to prevent water scarcity (articles 4.3 and 4.23) that are enforced with Environmental Permits (articles 5.1 and 5.24). Finally, articles 13.4 and 13.4b state that groundwater taxes based on the volume extracted can be imposed by provinces, which may differ for extraction activities in and outside of ground water protection areas used for drinking water production. Taken together, these articles in the Environment and Planning Act can be considered essential for the integration of the WFD provisions concerning water extraction and pricing on the national layer.

## The Netherlands Decision on the Quality of the Living Environment

The Decision on the Quality of the Living Environment provides command-and-control instruments on the national level for environmental issues such as water use and nature conservation (*Besluit Kwaliteit Leefomgeving*, 2024). As a first measure relating directly to drought, a national hierarchy in water allocation priorities during scarcity is determined in article 3.14, consisting of four levels: (1) the highest priority is to allocate water to ensure the stability of flood defenses, to prevent land subsidence and permanent damage to nature, followed by; (2) allocating water for drinking water and energy production; (3) allocating water for temporary irrigation of capital-intensive crops and industrial process water, and finally; (4) allocating water to other uses, including inland shipping, fisheries and less vulnerable agriculture and nature. Adding onto this, article 7.13 states that rules can be added to the water allocation hierarchy in Provincial Environment and Planning Regulations (*Omgevingsverordening*) for regional waters. Regional governments furthermore have to include provisions to preserve water conditions in Nature Network Netherlands areas (article 7.7), which are protected natural areas that include Natura 2000 areas, and provisions to ensure that Environmental Permits are only issued when WFD surface and ground waters are not adversely affected (article 7.11 and 7.12) and activities are compatible with water scarcity prevention objectives (article 8.22). Figure 4 shows ground and surface water bodies that are protected under the WFD and Figure 5 shows Natura 2000 and Nature Network Netherlands areas.

## The Netherlands National Environment and Planning Strategy

The National Environment and Planning Strategy (*Nationale Omgevingsvisie (NOVI)*, 2022) outlines the spatial planning and environmental management strategy for the Netherlands up to 2050. Droughts are explicitly addressed in the section on freshwater with non-binding vision statements (p. 137). The overarching strategy is to solve drought problems locally, to change land use to align with local water availability, to establish “a robust system” and to be transparent about the local water availability. The creation of Supplementary Strategic Supplies (*Aanvullende Strategische Voorraden*) for drinking water is also envisioned, as well as efforts to reduce water use of civilians and companies. Furthermore, a preferential order of action is put forward to prevent drought: (1) increasing water retention in the affected areas; (2) allocating water ‘smartly’, and; (3) accepting damages. Finally, the National Environment and Planning Strategy proposes the implementation of natural climate buffers, which are protective zones around natural areas designed to maintain adequate quantities of water during adverse climatic events. Apart from the section on freshwater, the section on agriculture in vulnerable areas addresses drought by referring to the use of CAP support to facilitate climate change adaptation (p. 149). The envisioned Supplementary Strategic Supplies are visualized in Figure 4, and the climate buffers are envisioned around the protected nature shown in Figure 5.

## The Netherlands National Strategic Plan CAP 2023-2027

The National Strategic Plan of the Netherlands (The Netherlands National Strategic Plan CAP 2023-2027, 2023) outlines estimates for the number and average amounts of subsidies across various intervention categories. Based on the “intervention logic” section (‘SO4’), five economic instruments in the CAP National Strategic Plan of the Netherlands (The Netherlands National Strategic Plan CAP 2023-2027, 2023) can be identified that address drought.

Eco-scheme payments (I.31) for crops that require less water, such as fiber crops and perennials, can be considered to address drought (Lampkin et al., 2020; Van Den Oever et al., 2023). The 2024 overview of eco-scheme payments shows that farmers in sandy soil regions in the East and South of the Netherlands currently receive € 720/ha for fiber crops, which is higher than in other parts of the country (€390) (*Punten en waarde eco-activiteiten 2024*, 2024). In the 2023 version of the eco-scheme payments, payments for fiber crops to farmers in sandy soil regions were €129, showing a significant increase in 2024. The National Strategic Plan reserves a total of €152 million per year for eco-scheme payments, which can increase to €355 million, depending on whether the Dutch government is legally permitted to transfer funds to the eco-scheme payments (p.302).

Subsidies are provided for investments in tangible (i.e., more efficient irrigation systems) and intangible assets, research and innovative production methods in the fruits and vegetables sectors that contribute to climate change adaptation (I.47). It is estimated that the number of subsidies will increase from 7 to 12 subsidies until 2028. The average subsidy is estimated to be €8,000,000 (p.305). As can be seen in Figure 5, fruits and vegetables farming is limited in Twente, and more prevalent in the North-East of the Vechtstromen region.

Payments for areas with natural and regulatory (i.e., WFD) constraints are provided to agricultural collectives (I.70). It is estimated that until 2028, the amount of land eligible for the Agricultural Nature and Landscape Management Subsidies will increase from 100,000 to 130,000 hectares, and that the subsidies will increase from €784.42/ha to €925.00/ha on average (p. 426).

Investments related to the Rural Development Fund are provided to farmers and non-agricultural businesses in rural areas for water retention measures such as widening water trajectories (I.73). It is estimated that 20 to 25 subsidies will be granted yearly with an average of €650,000 to non-agricultural businesses (p. 472), and 125 subsidies per year with an average of €125,000 to farmers (p.465).

Subsidies are offered to partnerships for integral area developments that aim to improve the hydrological conditions (I.77). The estimated number and average subsidy size vary, depending on the nature of the subsidized activity (i.e., plan development, execution). In total, €93,815,000 is expected to be invested in integral area development subsidies (p. 538).

Furthermore, harvest insurance against adverse climatic events is offered, where farmers receive compensation for the insured value if a damage threshold of 20% is met (I.76 for farmers in general, and I.47 for the fruits and vegetables sector). It is estimated that 4,388 beneficiaries will be compensated for an average of €4,034 (p.489).

## National Delta Program and Delta Plan Freshwater

The current Delta Program addresses drought in the Delta Plan Freshwater (*Deltaplan Zoetwater 2022-2027*, 2021), which reserves €200 million for investments in drought adaptation measures in Eastern sandy soil regions. The Delta Plan Freshwater currently reserves €137 million for investments in interventions in physical water system assets such as reprofiling water trajectories, implementing local dewatering and drainage measures and decoupling run-off from paved surfaces from the sewage system to increase infiltration. Another €6 million is reserved for investments in measures to promote efficient water use by land users, including soil enhancement measures and targeted irrigation systems. Finally, €55 million is reserved by the government for investments in land use change processes to create space for water retention and a smaller amount of €1,4 million is to be invested in the conversion of pine forests into heath or deciduous forests. As can be seen here, significant investments are to be made in the physical adaptation of land and water use systems to drought, which can be interpreted as an important step in drought adaptation, even though the investment plan is not binding and does not feature a detailed timeline. Furthermore, it should be clarified that these investments are used, among others, to fund the Freshwater Eastern Netherlands subsidies (*Zoetwater Oost-Nederland subsidies*) on the provincial layer, as further discussed in section 3.3. In terms of instrument types, the Delta Program measures can be interpreted as non-binding vision statements about investments in project-based (i.e., converting pine forests) and economic instruments (i.e., provincial subsidies). Pine forests in the region are shown in Figure 5.

## River Basin Management Plans, Programmes of Measures and National Water Program

River Basin Management Plans and Programmes of Measures mandated by the Water Framework Directive (Directive 2000/60/EC, 2000) are provided as part of the National Water Program in the Netherlands *(Nationaal Water Programma 2022-2027, 2022; Stroomgebiedbeheerplannen Rijn, Maas, Schelde en Eems* 2022 – 2027, 2022). The National Water Program explicitly addresses drought, albeit predominantly with abstract vision statements that concern increasing water use efficiency and water retention *(*p. 71-78 in *Nationaal Water Programma 2022-2027, 2022;* p. 119-121 in *Stroomgebiedbeheerplannen Rijn, Maas, Schelde en Eems* 2022 – 2027, 2022). Most concretely, the Programme of Measures (p.293 in *Nationaal Water Programma 2022-2027*, 2022) outlines several drought measures that center on research, water monitoring, improving hydrological models and a continuous development of management plans such as the River Basin Management Plans. A single project-based instrument can be identified here that concerns physical adaptation to drought: the canals in the Twente region, as highlighted in Figure 4, will be adapted to increase the robustness of water intake from the main water system. All of the drought measures in the Program of Measures are, however, non-binding and lack a detailed budget and time planning. The emphasis on research and the development of policies furthermore suggests that the policy process in the context of the National Water Program is still at an early stage.

## National Adaptation Strategy and National Implementation Program Climate Adaptation

The National Adaptation Strategy (*Nationale Adaptatie Strategie*, 2016), formulated by the government under the European Climate Law (Regulation (EU) 2021/1119, 2016) in 2016 did not address drought directly. However, the more recent follow-up document, the National Implementation Program Climate Adaptation (*Nationaal Uitvoeringsprogramma Klimaatadaptatie*, 2023), does address drought with non-binding vision statements for drought adaptation activities up to 2026 (p. 45 - 46). Similar to the National Water Program *(Nationaal Water Programma 2022-2027, 2022; Stroomgebiedbeheerplannen Rijn, Maas, Schelde en Eems* 2022 – 2027, 2022), these vision statements predominantly concern research (i.e., on alternative water supplies, water pricing, efficient water use), improving water monitoring and the development of drought measures, hereby suggesting that the policy process in the context of the National Adaptation Strategy is also at an early stage. Notable overlaps can furthermore be observed between the discussed non-binding national policies (i.e., Supplementary Strategic Supplies, Delta Plan Freshwater measures), which is due to fact that recent policies are based on policy studies that were initiated after the 2018 drought by the Drought Policy Platform (Beleidstafel Droogte, 2019) and the policy advisory board Project Team Sandy Soils (Projectteam Droogte Zandgronden Nederland, 2021).

## Provincial Environment and Planning Regulation of the Overijssel Province

In its current version, chapters 4 and 5 of the Provincial Environment and Planning Regulation of the Overijssel Province (*Omgevingsverordening Overijssel*, 2024) contain command-and-control instruments that relate to preserving water supplies and increasing the drought robustness of landscapes in the province. Chapter 4 of the Environment and Planning Regulation of the Overijssel Province (*Omgevingsverordening Overijssel*, 2024) provides provisions on the Environmental Plans (*Omgevingsplan*) that are formulated by municipalities and outline the requirements for spatial developments within their borders. Article 4.9 pertains that such requirements for spatial developments depend on local geospatial characteristics. Although areas relevant to drought adaptation (i.e., stream valleys) are demarcated as areas with “specific requirements” (article 4.9.3), these requirements are nevertheless unspecific and non-binding guidelines and do not explicitly address drought (*Catalogus Gebiedskenmerken Overijssel*, 2023). Article 4.13 furthermore pertains that Environmental Plans should only permit developments that contribute to climate-robust water and soil systems and to attaining water management objectives outlined in the Provincial Environment and Planning Strategy (*Provinciale Omgevingsvisie*). Articles 4.39 and 4.41 require Environmental Plans to only permit the development of new agricultural plots on sites near natural areas designated for "entrepreneurship with nature and water", as can be seen in Figure 5, if it strengthens the water system. Articles 4.57-4.66 implicitly address drought by requiring that provisions aimed at the maintenance or enhancement of ecological values are included in Environmental Plans for developments in protected and unprotected natural areas. A final drought measure in chapter 4 can be identified in article 4.88, which states that developments in water storage zones should not hinder water retention efforts or involve water-sensitive crops. Figure 4 shows that the areas for water storage are small and few in number.

Chapter 5 of the Environment and Planning Regulation of the Overijssel Province (*Omgevingsverordening Overijssel*, 2024) contains provisions on water management, particularly for water authorities. Most directly related to drought are the provisions on groundwater extraction presented in articles 5.14-5.20, requiring that Water Authority Regulations (*Waterschapsverordening*) only allow water extraction and infiltration volumes between 50,000 and 150,000 cubic meters per year if a permit has been issued or if the extraction activity is reported in advance. Permits for extraction volumes exceeding 150,000 cubic meters per year for industry and drinking water production are issued by the province, and extractions below 50,000 cubic meters are currently unregulated – which is permitted under the Water Framework Directive’s exemption on authorizing water extractions “which have no significant impact on water status”. Current water extraction activities are shown in Figure 6. As can be seen, water extraction activities for irrigation are less prevalent in the (drier) East of the study area and, more worrisome – yet understandable given the relatively high availability of groundwater – water extraction activities for industry and drinking water production are often located in close proximity to natural areas. Article 5.22 introduces deviations from national water allocation priorities formulated in the National Decision on the Quality of the Living Environment (*Besluit Kwaliteit Leefomgeving*, 2024) for the Vechtstromen region, adding water allocation to flush away salinization and pollutants to the fourth priority level and revising the fifth level almost entirely to prioritize water allocation to flush waterways in case of an acute risk for public health, to enable inland shipping, to maintain adequate water levels for arable farming and less vulnerable nature, to irrigate crops, and to flush less vulnerable nature, hereby omitting uses like fisheries, water recreation, energy production and non-essential drinking water production.

## Provincial Environment and Planning Strategy of the Overijssel Province

The Provincial Environment and Planning Strategy of the Overijssel province is (at the time of writing) not fully published yet. Therefore, the analysis is limited to the study of a foundation document (*Fundament voor de Nieuwe Omgevingsvisie,* 2022) and a concept version of the document (*Concept Nieuwe Omgevingsvisie*, 2024). Currently, the Environment and Planning Strategy distinguishes a number of spatial conditions that require different drought adaptation strategies. The strategy for areas that can be supplied with non-local water (i.e., that are close to primary water bodies such as rivers) concerns increasing water retention, reducing water use and widening water trajectories. Topographically higher moraines and sand ridges that cannot be supplied with non-local water (i.e., far removed from main water bodies) are to focus on reducing evapotranspiration, delaying run-off and increasing infiltration. In topographically lower areas that cannot be supplied with non-local water, the dewatering base should be raised and more space should be reserved to capture peak rainfall. In stream valleys that cannot be supplied with non-local water, drainage should be delayed and water trajectories should be lengthened by creating meanders. Non-spatially specific measures for areas that cannot be supplied with non-local water include reducing the demand for ground water extraction, increasing water retention and infiltration where possible and aligning land use with the water system. As can be seen in Figure 6, Twente region mainly consists of topographically higher areas and areas without water supply, hence implying a local strategy that centers on reducing evapotranspiration, delaying run-off and increasing infiltration.

## Fresh Water East-Netherlands subsidies

The Fresh Water East-Netherlands subsidies (*Zoetwater Oost-Nederland subsidies*) are part of the investment plan of the national Delta Plan Freshwater (*Deltaplan Zoetwater 2022-2027*, 2021). The legally binding rules for the subsidies are specified in article 2.5 of the Executive Order Subsidies of the Province of Overijssel (*Uitvoeringsbesluit subsidies Overijssel 2022*, 2024). Essentially, the subsidies are for municipalities, water authorities and the Netherlands Agricultural and Horticultural Association (*Land- en Tuinbouw Organisatie Nederland*) and they are provided for investments in the same interventions as those mentioned in the national Delta Plan Freshwater: land use change of pine forests, restoration of stream valleys, adjustable drainage and more efficient irrigation systems, among others.

## Delta Plan for Agricultural Water Management

The Delta Plan for Agricultural Water Management (*Deltaplan Agrarisch Waterbeheer Overijsselse Maatregelen*, 2023) provides subsidies to farmers to improve soil and water conditions. Subsidies for physical drought measures can cover up to 40% of the costs or up to a total of €10,000, whereas subsidies for advisory services can cover up to €1,500. Subsidies for drought measures are classified into six categories: (1) efficient irrigation systems and irrigation monitoring system; (2) water retention and drainage measures, such as the installation of weirs, shallowing ditches and creating wet banks; (3) increasing soil organic matter by reusing crop residue; (4) implementing run-off collection measures, such as infiltration ditches; (5) disseminating knowledge on, among others, sustainable land uses, and; (6) a miscellaneous category consisting of subsidies for advisory services on more conscious irrigation practices and for production losses incurred due to a change to climate robust land uses.

## Provincial Tax Regulation and Groundwater Taxes

Article 2 of the Provincial Tax Regulation (*Belastingverordening Overijssel*, 2024) contains the conditions for when ground water extraction charges are applied: a charge of €0.016 per cubic meter water extracted is applied to actors extracting more than 100,000 cubic meters per year, provided that these actors have a permit or reported the extraction activities. The volume of infiltrated water is deducted from the volume of water extracted.

## Provincial Nature Management Plan, Nature and Landscape Management Subsidies and Quality Impulse for Nature and Landscape Subsidies

The provincial layer addresses drought in the Provincial Nature Management Plan (*Natuurbeheerplan 2025 Provincie Overijssel*, 2024). Aimed at integrating the Birds and Habitats Directives, the WFD and the CAP on the provincial level, the Nature Management Plan contains non-binding vision statements on the management of protected nature and natural areas managed by farmers. In terms of drought adaptation, vision statements currently emphasize increasing water retention in stream valleys and infiltration areas, and changing land use from corn to cereals that require less water. These vision statements are supported by two voluntary economic instruments that target specific areas: the Nature and Landscape Management (*Subsidieregeling natuur- en landschapsbeheer Overijssel 2024*, 2024) and the Quality Impulse for Nature and Landscape (*Subsidieregeling kwaliteitsimpuls natuur en landschap Overijssel 2024*, 2024) subsidy schemes. Nature and Landscape Management subsidies are granted for nature management practices of agricultural collectives and is provided for drought measures such as increasing water retention in stream valleys and infiltration areas, land use change (i.e., corn to cereals), and the maintenance of appropriate water levels **(**section **K01.01)**. As can be seen in Figures 5 and 6, cornfields and stream valleys are widespread throughout the region, which implies that a large area is eligible for these subsidies, which in turn raises concerns about the potential subsidy burden if (hypothetically speaking) all land users were to be supported. The Quality Impulse for Nature and Landscape subsidies are granted to land users to enhance natural qualities, for example by converting agricultural land to nature, and are not explicitly aimed at drought adaptation and only concern a small number of areas that are too small to be visible in a map.

## Water Management Regulation of the Vechtstromen Water Authority

The Water Authority Regulation (*Waterschapsverordening Waterschap Vechtstromen*, 2024), explicitly addresses drought challenges with binding command-and-control instruments in articles 1 and 4. Article 1.14 states that Environmental Permits (*Omgevingsvergunning*) can only be issued on the condition that the activities align with legislation (i.e., WFD) and strategies (i.e., National Water Program, the Water Authority’s Water Management Plan) to prevent water scarcity. Next, article 1.17 concerns water extraction and dewatering prohibitions if there is a severe case of water scarcity, even if this implies deviation from issued Environmental Permits. Article 3.31 seems to refer to the European Union Water Reuse Regulation and Urban Wastewater Treatment Directive by stating that wastewater should be reused before discharging it. Article 4 provides several provisions on water extraction and infiltration: the obligation to report water extraction (articles 4.4, 4.5, 4.11 and 4.14), irrigation bans in a 200-meter radius around ground water-dependent nature (4.9) and the requirements of Environmental Permits for water extraction (article 4.6, 4.7 and 4.16). The provisions on permits for, and reporting of, water extraction activities are similar to the provisions provided in the Provincial Environmental Regulation (*Omgevingsverordening Overijssel*, 2024). However, they provide more detail for water extraction activities for the purposes of irrigation and soil remediation in terms of the allowed pump capacity and extraction volumes over a 30-day period.

## Water Management Plan of the Vechtstromen Water Authority

The Water Management Program of the Vechtstromen Water Authority (*Waterbeheerprogramma 2022-2027 Waterschap Vechtstromen*, 2021) for the period between 2022 and 2027 outlines four “tracks” of measures: (1) regulation, (2) management, (3) financial investments and (4) collaboration with water users and other governance layers. The measures are summarized in the next paragraphs and concern existing measures that the Vechtstromen Water Authority has established through participation processes and aims to continue implementing. However, the measures are non-binding, and neither budgets nor timelines, apart from a five-year horizon, are presented in the document. The measures should thus predominantly be interpreted as vision statements, although the measures in the investment and collaborative tracks show elements of project-based, informational and cooperative instruments.

The regulation track of the Water Management Program (p. 41 in *Waterbeheerprogramma 2022-2027 Waterschap Vechtstromen*, 2021) contains merely one measure that is currently in place, which concerns flood safety. However, there are a number of drought policies that are to be put on the agenda in the future. These future policies mainly concern the recalibration of ground and surface water extraction policies, drainage policies and the development of an integral ground water policy in relation to climate change and protecting drinking water supplies.

Nine measures are currently in place in the management track that address drought challenges (p. 44 in *Waterbeheerprogramma 2022-2027 Waterschap Vechtstromen*, 2021). The first three of these measures concern the installation, automation and climate adaptive operation of weirs. The fourth measure concerns the replacement of waterworks in general to increase their climate robustness. Then, three measures concern research on: the number and volume of ground and surface water extractions; an accelerated renovation of weirs to enhance climate resilience and; the value of telemetry systems for remote water system monitoring. The eighth measure concerns an adjustment of the assessment framework for the size and shape water bodies to align with climate robustness objectives. Finally, the ninth measure is to align measures in operational water level management with target water levels. Important to highlight, is that one measure in the management track seems to be contradicting with national and provincial drought policy objectives, as well as with the long-term ambitions of the Water Authority itself: it is explicitly stated that “water levels will remain aligned with land use” in flat and sloped areas, rather than adopting the opposite approach. Essentially, this implies that water levels can remain low to ensure sufficient drainage to accommodate the desired land uses, regardless of whether this increases the drought vulnerability of the landscape. In paragraph 3.2 of the Water Management Plan, it is furthermore stated that water levels are determined together with land users, which might imply that concerns exist among land users with regards to land use change.

In the investment track, four existing measures address drought (p. 47 in *Waterbeheerprogramma 2022-2027 Waterschap Vechtstromen*, 2021). First, the Water Authority aims to invest in shallowing and lengthening water trajectories by creating meanders. Second, investments will be made in the automation of weirs to enhance flexibility in adjusting water levels. Third, the water authority aims to address bottlenecks in the supply of non-local water from the regional main water system. Fourth, the Water Authority will make investments to separate wastewater and rainwater sewage systems in urban areas, which will facilitate infiltration and re-use of rainwater.

Lastly, the Water Management Program formulates six information-based and cooperative instruments in the collaboration track (p. 50 in *Waterbeheerprogramma 2022-2027 Waterschap Vechtstromen*, 2021). The information-based instruments mentioned in this track focus on increasing awareness about climate robust land use, on promoting the use of Delta Plan Agricultural Water Management subsidies (*Deltaplan Agrarisch Waterbeheer Overijsselse Maatregelen*, 2023), on stimulating the installation of adjustable dewatering measures and on providing information about water retention measures using Delta Plan Agricultural Water Management and Freshwater East Netherlands subsidies (*Deltaplan Agrarisch Waterbeheer Overijsselse Maatregelen*, 2023; *Zoetwatervoorziening Oost-Nederland subsidies* in *Uitvoeringsbesluit subsidies Overijssel 2022*, 2022). The cooperative instruments in this track aim to seek alignment with provinces and drinking water companies on securing a climate robust drinking water supply and with municipalities on establishing joint implementation agendas.

## Water Vision of the Vechtstromen Water Authority

Drought is one of the main challenges addressed in the Vechtstromen Water Authority Strategy (*Watervisie 2050 Waterschap Vechtstromen*, 2020). Three long-term non-binding vision statements can be identified that concern future drought adaptation measures: (1) adapting water and land use systems to restore the balance between dewatering and water retention; (2) increasing the water retention capacity of the soil by, among others, increasing the height of the weirs, reserving more space for water retention, reducing the dewatering capacity, and decoupling rain water and waste water systems to facilitate water reuse, and (3); making the water system attractive for recreational uses to increase awareness about the value of water and the social acceptability of water system interventions. The strategy does not include any budgets or time planning, except for a 30-year time horizon.

## References

Bastmeijer, K., van Rijswick, M., & Verschuuren, J. (2021). *Verdroginging Brabant: Een Europees Rechtelijk Perspectief*. Tilburg University.

Beleidstafel Droogte. (2019). *Nederland beter weerbaar tegen droogte*.

*Besluit Kwaliteit Leefomgeving*. (2024).

*Catalogus Gebiedskenmerken Overijssel*. (2023).

*Consolidated Version of the Treaty on the Functioning of the European Union*. (2016).

Copetti, D., & Erba, S. (2023). A bibliometric review on the Water Framework Directive twenty years after its birth. *Ambio*. https://doi.org/10.1007/s13280-023-01918-0

*Deltaplan Agrarisch Waterbeheer Overijsselse Maatregelen*. (2023).

*Deltaplan Zoetwater 2022-2027*. (2021).

*Directive (EU) 2024/3019 of the European Parliament and of the Council of 27 November 2024 concerning urban wastewater treatment (recast) (Text with EEA relevance)*. (2024).

*European Climate Law, Regulation (EU) 2021/1119* (2nd ed.). (2016). Hart Publishing Ltd. https://doi.org/10.5040/9781782258674

Heyl, K., Döring, T., Garske, B., Stubenrauch, J., & Ekardt, F. (2020). The Common Agricultural Policy beyond 2020: A critical review in light of global environmental goals. *Review of European, Comparative & International Environmental Law*. https://doi.org/10.1111/reel.12351

Lake, P. S. (2003). Ecological effects of perturbation by drought in flowing waters. *Freshwater Biology*, *48*(7), 1161–1172. https://doi.org/10.1046/j.1365-2427.2003.01086.x

Lal, R. (2020). Soil organic matter and water retention. *Agronomy Journal*, *112*(5), 3265–3277. https://doi.org/10.1002/agj2.20282

Lampkin, N., Stolze, M., Meredith, S., de Porras, M., Haller, L., & Meszaros, D. (2020). *Using Eco-schemes in the new CAP: a guide for managing authorities.* IFOAM EU, FIBL and IEEP.

*Nationaal Uitvoeringsprogramma Klimaatadaptatie*. (2023).

*Nationaal Water Programma 2022-2027*. (2022).

*Nationale Adaptatie Strategie*. (2016).

*Nationale Omgevingsvisie (NOVI)*. (2022).

*Omgevingsverordening Overijssel*. (2024).

*Omgevingswet*. (2024).

Projectteam Droogte Zandgronden Nederland. (2021). *Droogte in zandgebieden van Zuid-, Midden- en Oost-Nederland*.

Provincie Overijssel. (2022). *Fundament voor de Nieuwe Omgevingsvisie*.

Provincie Overijssel. (2024). *Concept Nieuwe Omgevingsvisie Overijssel*.

*Punten en waarde eco-activiteiten 2024*. (2024).

*Regulation (EU) 2021/2115 establishing rules on support for strategic plans to be drawn up by Member States under the common agricultural policy (CAP Strategic Plans) and financed by the European Agricultural Guarantee Fund (EAGF) and by the European Agricultural Fund for Rural Development (EAFRD) and repealing Regulations (EU) No 1305/2013 and (EU) No 1307/2013*. (2024).

*Regulation (EU) 2024/1991 of the European Parliament and of the Council of 24 June 2024 on nature restoration and amending Regulation (EU) 2022/869*. (2024).

Reich, P., & Lake, P. S. (2015). Extreme hydrological events and the ecological restoration of flowing waters. *Freshwater Biology*, *60*(12), 2639–2652. https://doi.org/10.1111/fwb.12508

Sarremejane, R., Messager, M. L., & Datry, T. (2022). Drought in intermittent river and ephemeral stream networks. *Ecohydrology*, *15*(5), e2390. https://doi.org/10.1002/eco.2390

*Stroomgebiedbeheerplannen Rijn, Maas, Schelde en Eems 2022 – 2027*. (2022).

*The Netherlands National Strategic Plan CAP 2023-2027*. (2023).

Thieme, M., Birnie-Gauvin, K., Opperman, J. J., Franklin, P. A., Richter, H., Baumgartner, L., Ning, N., Vu, A. V., Brink, K., Sakala, M., O’Brien, G. C., Petersen, R., Tongchai, P., & Cooke, S. J. (2024). Measures to safeguard and restore river connectivity. *Environmental Reviews*, *32*(3), 366–386. https://doi.org/10.1139/er-2023-0019

*Uitvoeringsbesluit subsidies Overijssel 2022*. (2024).

Van Den Oever, M., Vural Gursel, I., Elbersen, W., Kranendonk, R., Michels, R., & Smits, M.-J. (2023). *Regional supply of herbaceous biomass for local circular bio-based industries in the Netherlands*. Wageningen Food & Biobased Research. https://doi.org/10.18174/630159

*Water Framework Directive (2000/60/EC)*. (2000).

*Waterbeheerprogramma 2022-2027 Waterschap Vechtstromen*. (2021).

*Waterschapsverordening Waterschap Vechtstromen*. (2024).

*Watervisie 2050 Waterschap Vechtstromen*. (2020).
